# Glial-restricted progenitor cells: a cure for diseased brain?

**DOI:** 10.1186/s40659-024-00486-1

**Published:** 2024-03-12

**Authors:** Piotr Rogujski, Barbara Lukomska, Miroslaw Janowski, Luiza Stanaszek

**Affiliations:** 1 NeuroRepair Department, Mossakowski Medical Research Institute, Polish Academy of Sciences, 02-106 Warsaw, Poland; 2 Department of Diagnostic Radiology and Nuclear Medicine, University of Maryland, Baltimore, MD USA

**Keywords:** Glial-restricted progenitor, Oligodendrocyte precursor cell, Oligodendrocyte, Myelin, Pre-clinical study, Cell therapy

## Abstract

The central nervous system (CNS) is home to neuronal and glial cells. Traditionally, glia was disregarded as just the structural support across the brain and spinal cord, in striking contrast to neurons, always considered critical players in CNS functioning. In modern times this outdated dogma is continuously repelled by new evidence unravelling the importance of glia in neuronal maintenance and function. Therefore, glia replacement has been considered a potentially powerful therapeutic strategy. Glial progenitors are at the center of this hope, as they are the source of new glial cells. Indeed, sophisticated experimental therapies and exciting clinical trials shed light on the utility of exogenous glia in disease treatment. Therefore, this review article will elaborate on glial-restricted progenitor cells (GRPs), their origin and characteristics, available sources, and adaptation to current therapeutic approaches aimed at various CNS diseases, with particular attention paid to myelin-related disorders with a focus on recent progress and emerging concepts. The landscape of GRP clinical applications is also comprehensively presented, and future perspectives on promising, GRP-based therapeutic strategies for brain and spinal cord diseases are described in detail.

## Introduction

The central nervous system (CNS), traditionally divided into the brain, spinal cord, and retina, is composed of neural tissue consisting of neuronal and glial cells. Glia is composed of astrocytes, oligodendrocytes, ependymocytes, and microglia.

Glial-restricted progenitors (GRPs), glial progenitor cells (GPCs), oligodendrocyte progenitors, or precursor cells (OPCs), or NG2-cells [38, 111], represent approximately 5% of all cells of the central nervous system and are the primary source of myelinating oligodendrocytes in the CNS [22]. Despite some discrepancies, in the following review article, the terms “GRPs” and “OPCs” shall be defined as did the authors of a cited publication. However, for clarity, please refer to Chapter 2 (Nomenclature duality: The relationship between GRPs and OPCs) below.

Due to the enormous complexity of neuronal circuits, synaptic architecture and interactions between various cells of the CNS brain functioning is still not fully understood, and so are the means to treat pathologies thereof. As of 2017, the annual cost of treating neurological diseases reached $800 billion in the US alone, with some orphan illnesses still lacking effective remedies [40]. Since oligodendrocytes are the sole source of myelin across the CNS, their pathologies and subsequent demyelination cause many diseases in children, those include various leukodystrophies, including Pelizaeus-Merzbacher disease (PMD) or Krabbe disease (KD), and in adults—pathologies such as amyotrophic lateral sclerosis (ALS), multiple sclerosis (MS), Huntington’s disease (HD), or spinal cord injury (SCI). They are also linked to Alzheimer’s disease (AD) and schizophrenia [38]. Given that most of these pathologies are in some way related to glial dysfunction, transplantation of exogenous cells with glia-generating potential could be a new, promising therapeutic approach. Such grafts could compensate for glial deficits and act as cellular mediators stimulating endogenous regeneration and replacements for defective glia.

GRPs can differentiate into astrocytes and oligodendrocytes in vitro and in vivo [88]. Meanwhile, differentiation into neurons was not reported even upon migration into neurogenic environments [50].

## Nomenclature duality: the relationship between GRPs and OPCs

At this stage, the Reader may already seem confused with the GRPs/OPCs nomenclature duality. Unfortunately, there are still contradicting opinions when defining a clear relationship between glial GRPs and OPCs based on the past literature. As shown by our group GRPs are multipotential cells with the potential to only generate cells from the glial lineage. These include oligodendrocyte progenitor cells (OPCs) which give rise to oligodendrocytes, and astrocyte progenitor cells (APCs) generating type 1 and type 2 astrocytes [146]. Martins-Macedo et al. attempted to list all the glial precursors, demonstrating some distinctions between oligodendrocyte progenitor cells/oligodendrocyte and type-2 astrocyte, motoneuron-oligodendrocyte precursors, astrocyte restricted precursors/astrocyte precursor cells, white matter progenitor cells, and glial restricted precursor cells (GRPs) [94].

In a landmark study, Weng et al. attempted to delineate the diversity and cell fate determinants of glial progenitors on a transcriptomic level by targeted single-cell RNA sequencing. By adopting unsupervised clustering using t-distributed stochastic neighbor embedding (t-SNE), the authors found that in the developing mouse cortex, OPCs exhibit a fate-restricted continuum encompassing an intermediate, “pri-OPC” population closely resembling adult activated NSCs (PPP1R14B^+^, ASCL1^+^, BTG2^+^, HES6^+^) and a finally-committed OPC cluster (PDGFRA^+^, CSPG4^+^). Moreover, a fraction of both pri-OPCs and OPCs express cell cycle-related genes, which indicates that they remain proliferative during early oligodendrogenesis [170]. To provide more insight into the transitions between various oligodendrocyte-generating progenitors, Marques et al. performed bulk and single-cell RNA sequencing on the forebrains and spinal cords of E13.5 and P7 mice. In their findings, the group observed that PDGFR^+^ cells within the central nervous system display substantial heterogeneity. However, there seems to be a convergence in spatial and temporal transcriptional profiles during the shift from embryonic pre-OPCs to OPCs in development. OPCs emerging from different areas within the embryonic germinal zones eventually exhibit considerable similarity [91]. One may speculate that these cells might demonstrate a transitional state, either differentiating exclusively toward restricted OPCs (“pre-OPCs”) or, alternatively, they might be bi-potential progenitors retaining bona fide glia-generating potential (GRPs). However, as already shown, some authors use the names: GRPs and OPCs synonymously [39], often focusing on the myelination process as an oligodendrocyte function. Perhaps some generalizations were made during these studies due to limited research methodology or simplicity. Meanwhile, multipotential GRPs defined explicitly as oligodendrocyte and astrocyte-generating cells would be promising therapeutic cells for transplantation since today, apparent cooperation is demonstrated between glial cells in the brain, such as with astrocytes supporting oligodendrocyte survival and myelination [157]. Furthermore, there is also an evident glial component in many neurodegenerative diseases [141]. Therefore, we believe setting new therapeutic trends based on such up-to-date results would be advisable. One such idea would be the global glia replacement which tackles whole-brain events, such as global dysmyelination [146, 166]. In this review, we will keep the same cell terminology as used in the author’s original texts. However, for future clarity, we urge the scientific community to avoid further nomenclature ambiguity and treat OPCs as a progeny of GRPs.

## Characteristics of GRPs

The first step in utilizing the therapeutic potential of a given cell is to familiarize with its ontogenesis, phenotype, and functions. Below is the review of GRPs' fetal development providing insight into their endogenous niches, unique morphological and molecular identity by which they are identified, and the wide range of functions they can perform after exogenous delivery into the adult CNS.

### Ontogenesis

Following gastrulation, during ectodermal development, neuroepithelial cells generate and settle along the neuraxis within the ventricular subependyma where primary neural stem cells (NSCs) are forming, giving rise to neurons, astrocytes, radial glia, and GRPs. GRPs start migrating and eventually reside throughout the CNS [138].

The current understanding of human GRP (hGRP) ontogenesis is drawn partially from rodent studies. In mice, the development of GRPs occurs in three distinct waves with apparent, albeit subtle, differences between the brain and spinal cord. In the brain, the first wave occurs on embryonic day 12.5 (E12.5) in the ventral neural tube and is driven by a sonic hedgehog (Shh) derived from the ventral floor plate. Shh is one of the key signaling molecules involved in the regulation of morphogenesis during embryonic development and is necessary for expressing two GRP stage-specific transcription factors: Olig1 and Olig2, which, in turn, control the expression of key genes involved in oligodendrocyte specification and maturation [161]. GRPs form mainly within the medial ganglionic eminence and start migrating throughout the developing forebrain. Later, the second wave occurs on E15.5 from the lateral ganglionic eminence under the transcriptional control of Gsx2 and again progresses throughout the cortex. Finally, the third wave occurs neonatally and postnatally from the subventricular zone (SVZ). Driven by Emx1, these third-wave GRPs migrate throughout the brain and mix with GRPs derived at earlier stages [63].

Similarly, the first wave in the spinal cord is controlled by ventrally-derived Shh. The second wave occurs in the dorsal neural tube. It is transcriptionally regulated by Ascl1 and Dbx1 and requires upregulation of fibroblast growth factor (FGF) signaling and downregulation of bone morphogenetic protein (BMP) signaling. FGF functions via activation of its tyrosine kinase receptors which leads to modulation of cellular proliferation, migration, and differentiation, whereas BMP, being a member of the transforming growth factor beta superfamily, plays a crucial role in regulating apoptosis during embryonic development [99, 149]. The third and final wave of GRP generation in the spinal cord occurs neo- and postnatally. Origins are unclear: those GRPs might derive from progenitors remaining within the central canal or from precursors dispersed throughout the parenchyma [39]. Notably, cells originating during the first wave are considered redundant and may be postnatally eliminated; however, they can survive and replace GRPs born in later stages if necessary [63].

**Fig. 1:**
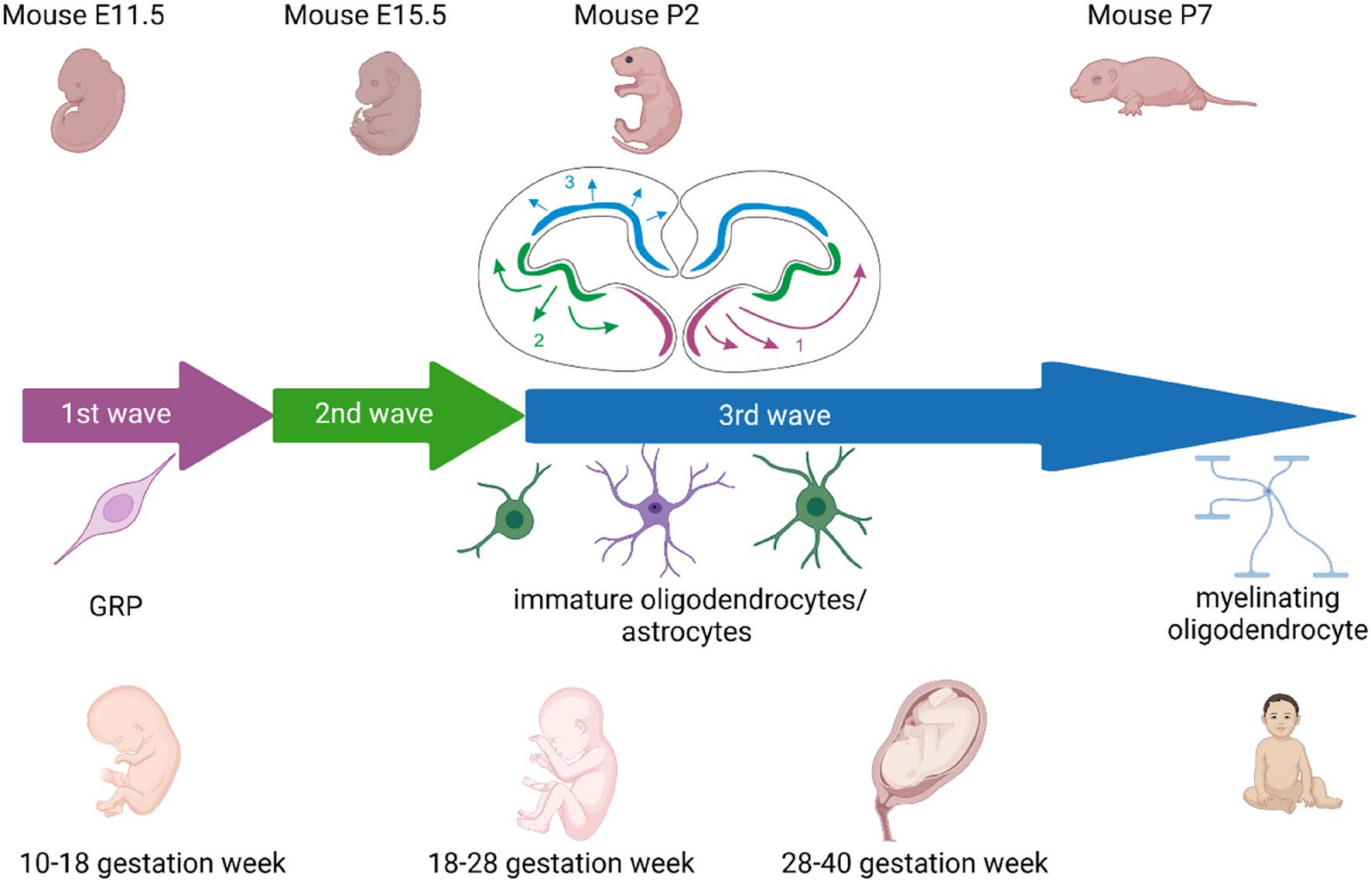
Comparison between different stages of neurodevelopment in mice and humans from the context of glial-restricted progenitors (GRPs)

Our detailed knowledge about hGRP ontogenesis is limited. However, as demonstrated in a landmark work by Sim and colleagues using flow cytometry, it is known that during neural development of the human forebrain, glial-restricted progenitors with oligodendrocyte-generating potential are detectable from 16–18 weeks of gestation (Fig. 1) [142]. Meanwhile, recently, Fu et al. have shed more light on the development of glial progenitors using single-cell RNA sequencing. The authors have shown that the population of EGFR^+^ cells contains a large fraction of progenitors from different lineages and that during the development of the human cerebral cortex, the expression of EGFR drastically increases at the start of gliogenesis (after gestational week 20). Moreover, the fraction of glial progenitors was markedly enriched after EGFR sorting during the switch from neurogenesis to gliogenesis [32]. Postnatally, GRPs can be found throughout the entire adult CNS, constituting around 8–9% of the whole white matter and 2–3% of the grey matter cell pools. This makes them the fourth-largest population of glial cells after astrocytes, microglia, and oligodendrocytes [22].

### Phenotype

**Fig. 2:**
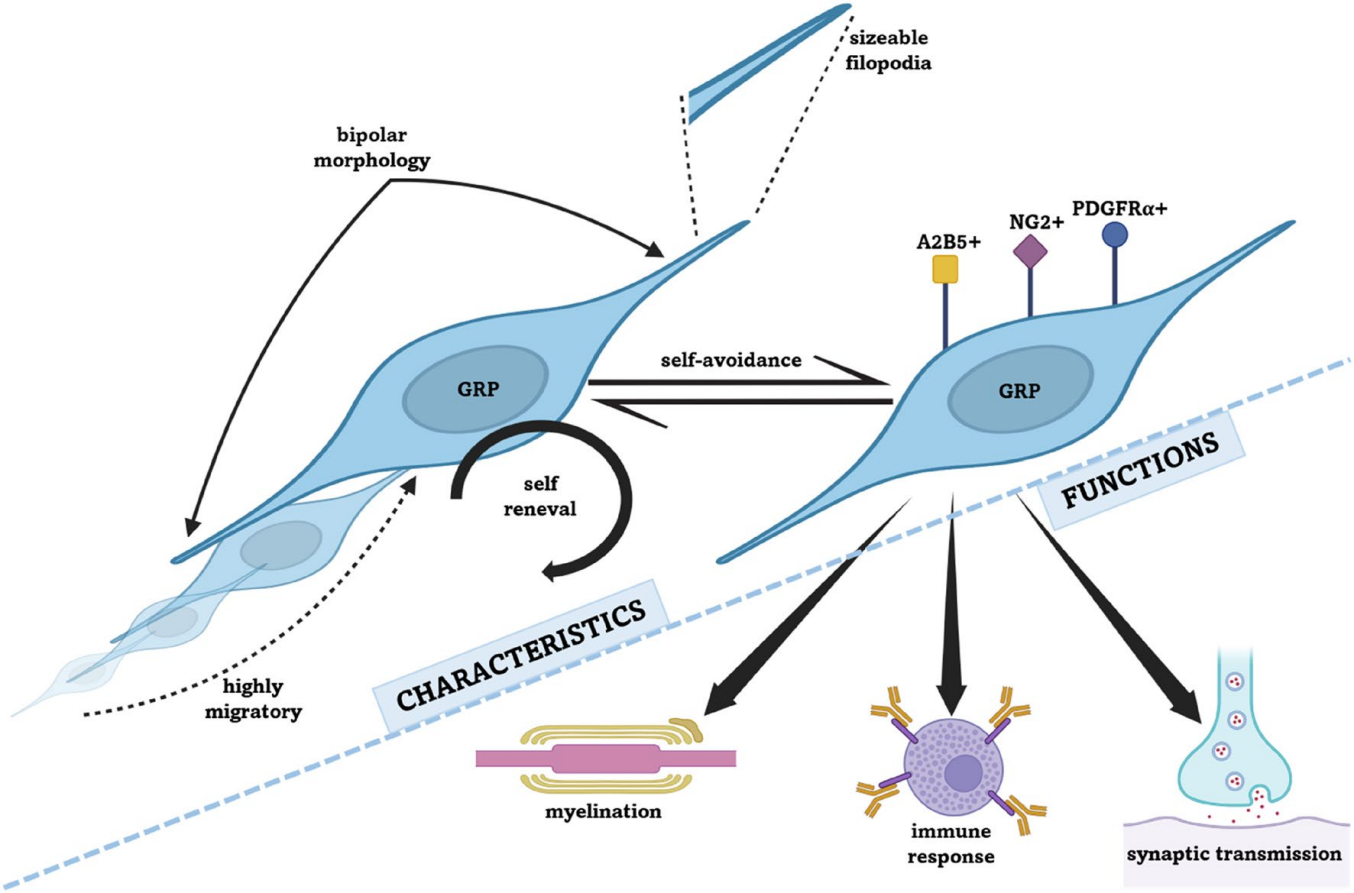
Characteristics and functions of glial-restricted progenitor cells (GRPs)

The origin of studies on glial progenitor development dates back to the 1980s when Raff and colleagues described the oligodendrocyte-type-2-astrocytes (so-called “O-2A” cells) in the rat optic nerve. These newly discovered entities could generate cells resembling astrocytes or oligodendrocytes in rats [128] and neurons in humans [116]. With their differentiation capabilities, bi-potential morphology, and high mitotic activity, GRPs were identified by one of their membrane epitopes–neural/glial antigen 2 (NG2), also known as chondroitin sulfate proteoglycan type 4 (CSPG4). This integral membrane protein is expressed in most non-neural cells in the CNS, is implicated in cell migration, proliferation, and modulation of neural plasticity, and contributes to the dynamic regulation of the neural microenvironment [128]. Another vital characteristic of neuronal and glial progenitors is the expression of A2B5. This surface ganglioside affects cell adhesion, migration, and differentiation during neuronal development and myelination, and is widely adopted in antibody-based cell purification assays [116, 129]. It is, however, of note that neither NG2 nor A2B5 are expressed exclusively on the GRPs’ surface. As mentioned, A2B5 is also expressed on neuronal progenitors, whereas NG2 can be found on pericytes [120]. A vital characteristic specific to GRPs is the expression of platelet-derived growth factor α receptor (PDGFαR), also known as CD140a. Activated by its ligand PDGFα, PDGFαR plays a central role in cellular processes like cellular migration and proliferation, particularly in the context of embryonic development, and is currently regarded as the only antigen expressed exclusively on GRPs of human CNS and is, therefore, among the most suitable targets for GRPs identification [142] (Fig. 2). Additionally, developed GRPs restricted only to oligodendrocytic lineage can be recognized by anti-O4 antibodies [144].

GRPs are highly migratory and maintain their unique territories through self-avoidance. They extend sizeable filopodia, which retract following contact with extensions from the same or adjacent GRP cell (Fig. 2). Due to a proliferative phenotype, they can continuously restore their pool, migrate to and settle within the sites of focal injury, and partake in regeneration. Those characteristics account for GRPs’ extraordinary capacity to maintain glial homeostatic balance [53], translating into the potential basis for cell replacement therapy.

### Functions

GRPs are the primary source of new oligodendrocytes in the adult brain. In turn, oligodendrocytes produce myelin, a specialized membrane consisting primarily of proteins and fatty acids. Myelin wraps around the nerve axon in a spiral fashion and provides electrical insulation, thus facilitating signal transmission across the nervous system [130]. Loss of myelin sheath is commonly attributed to numerous neural diseases and axonal damage. Although spontaneous remyelination with endogenous oligodendrocytes occurs, the restored myelin layer is usually thinner and provides weaker signal conduction [14, 143]. Aside from their myelinating activity, oligodendrocytes also play a vital role as metabolic supporters for neurons through lactate secretion [75]. Interestingly, GRPs can also generate Schwann cells in the CNS under certain conditions [190]. In specific diseases and experimental conditions, GRPs can generate type-1 and type-2 astrocytes, yet in modest numbers, incomparable to those generated via the direct proliferation of existing ones [159, 190]. Despite that, some works revealed the beneficial effects of transplanted, more developed glial-derived astrocytes (GDAs) from rats and humans on axonal growth and neuroprotection [20, 21]. It is worth noting that, in in vitro conditions and after reintroduction to the brain via transplantation, adult hGRPs can also give rise to neurons [116]. It was proven that neurons could differentiate from a small subset of rodent GRPs without external manipulation, yet in a relatively modest fashion [42, 133]. GRPs can establish synaptic transmission with GABAergic and glutamatergic neurons, possibly affecting the activity of neuronal circuits [12, 84].

Recent studies have shown GRPs' ability to be involved in the immune response by expressing MHC class II, acting as antigen-presenting cells, and activating effector and memory CD4^+^ T-cells [27]. Therefore, the range of potential therapeutic utilities for GRPs is truly compelling (Fig. 2).

## Sources of GRPs

**Fig. 3:**
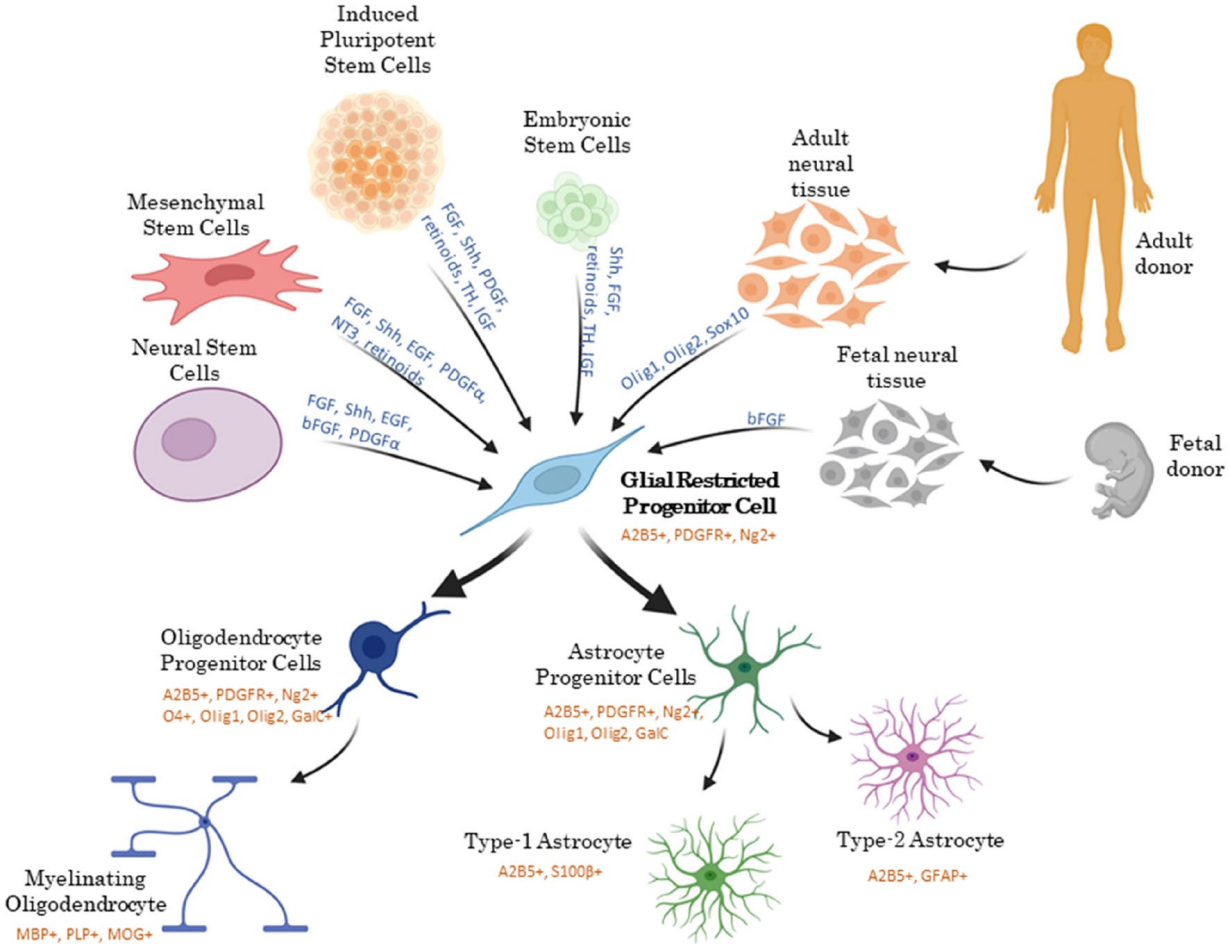
Sources of glial-restricted progenitor cells and their progeny

The second step in utilizing cells’ therapeutic potential is finding their most safe, reliable, and efficient source. Therapeutic GRP sources should be easily accessed in an appropriate quantity and present a significant potential for glial differentiation. In addition, the derivation protocol should be relatively prompt and affordable. Finally, the procedure should not raise ethical concerns [37, 38, 119]. Various sources of GRPs have been described, including direct isolation from embryonic, fetal, or post-natal neural tissues, direct- or indirect cellular reprogramming from somatic cells (induced pluripotent stem cells; iPSCs), or targeting other endogenous stem cells (Fig. 3). Below is a review of the most commonly utilized sources of therapeutic GRPs considering their availability, efficiency, and safety of the protocol.

### Embryonic stem cells

Embryonic stem cells are pluripotent cells derived from embryos' undifferentiated inner cell mass at the blastocyst developmental stage. Their pluripotency is defined as an intrinsic ability to differentiate into cells of all three germ layers: endoderm, mesoderm, and ectoderm. ESCs are also capable of self-replicating indefinitely, making them a potent source of cells for scientific and therapeutic purposes [93]. As shown by Brüstle and colleagues, GRPs can be derived in vitro from human embryonic stem cells directly. After aggregating into embryoid bodies (EBs; three-dimensional aggregates of pluripotent cells) and sequentially culturing hESCs in vitro in a defined medium containing, firstly, basic fibroblast growth factor 2 (FGF2); secondly, FGF2 and epidermal growth factor (EGF), and thirdly, FGF2 and platelet-derived growth factor (PDGF), proliferating cells with bipolar morphology, immunoreactive to A2B5 antibody, started to form. Eventually, when the growth factors were removed, cells differentiated into oligodendrocytes and astrocytes, confirming their bipotential phenotype [15].

Numerous other studies also proved the successful generation of hOPCs from hESCs. Most of these works rely on recapitulation of embryonic development, where functional glial progenitors are generated from pluripotent stem cells based on sequentially delivered signals. These include Shh, FGFs, retinoids, insulin growth factor (IGF), and thyroid hormone (TH). Nistor et al. obtained over 80% efficiency in generating oligodendrocyte precursors after 42 days of Matrigel-based hESCs culture [113]. Other groups utilized feeder layers to culture ESCs, including mouse embryonic fibroblasts (MEFs) [35, 51, 54, 61] and more clinically-relevant, human foreskin fibroblasts (HFFs) [52, 150, 151]. Most of these works delivered hOPCs from ESCs between 40 and 90 days, with 80–90% efficiency. Some modern approaches for deriving hOPCs from ESC included utilizing synthetic growth surfaces, such as vitronectin-derived synthetic peptide acrylate [83], and hydrogel-based, 3D cell culture systems [134] for a more scalable, clinically-relevant strategy.

It is, however, worth noting that despite their proven potential, hESCs isolation still requires sacrificing a living embryo, which again carries a significant ethical burden. Moreover, their unlimited proliferating potential creates a substantial risk of uncontrollable tumor formation after transplantation. Finally, ESC-based cell therapy still poses the risk of allograft rejection, imposing immunosuppression in transplantation recipients [172].

### Fetal and post-natal neural tissue

Initially, GRPs were isolated from rat primary cell culture. Using fresh E13.5 rat spinal cords, a 6-day neuroepithelial cell culture was established, followed by anti-A2B5 monoclonal antibody-based immunopanning. Isolated cells demonstrated high (> 90%) viability and both astrocyte- and oligodendrocyte differentiation potential [129]. Further studies also proved the successful generation of GRPs from adult human brain tissue after resection, where fluorescence-activated cell sorting (FACS) based on CNP2 promoter generated a small (< 0.5%) population of bipotential A2B5^+^ positive hGRPs [135]. Other works have shown derivation from adult human tissue GRP cells with even broader lineage potential of generating not only astrocytes and oligodendrocytes but also neurons [116]. Finally, hGRPs were also derived from human fetal tissue [77, 165, 173, 174]. However, to increase the purity of gliogenic hGRPs, the other selection was required. With A2B5-positive selection, PDGFαR epitope CD140a was also targeted, generating a small fraction of more molecularly defined, self-renewing bipotential progenitors with astrocytic and oligodendrocytic differentiation potential [142]. It is, however, worth mentioning that endogenous GRP cells are not all necessarily homogenous. In fact, from a molecular standpoint, they represent a substantially heterogeneous pool in rodents [92] and humans. Moreover, when adult and fetal GRPs were compared, three separate subsets of hGRPs could be distinguished: I. A2B5^+^, O4^−^, MOG^−^; II. A2B5^+^, O4^+^, MOG^−^; III. A2B5^−^, O4^+^, MOG^−^ in the fetal brains and: I. A2B5^+^, O4^−^, MOG-; II. A2B5^−^, O4^+^ (with low expression profile), MOG^−^; III. A2B5^+^, O4^+^ (with high expression profile), and MOG^+^ in GRPs isolated from the adult. Indeed, adult hGRPs demonstrate low to undetectable expression of miRNAs highly expressed in O4^−^ fetal GRPs [76]. Also, from an ethical standpoint, fetal neural tissue is still not a feasible source of cells as it requires terminating the pregnancy. Meanwhile, GRPs from adult human tissue can only be directly obtained via resection, which is unjustifiable unless accompanying reasons, such as tumor surgery, occur. Therefore, although adult and human fetal tissues are a proven source of therapeutic GRPs, they do not overcome ethical issues related to the isolation of cells from living donors nor deliver cells with sufficient yield and homogenous phenotype.

### Induced pluripotent stem cells

Induced pluripotent stem cells (iPSCs) are a relatively new source of ESC-like cells with pluripotent potential. Initially discovered by Yamanaka and Takahashi in 2006, the iPSC technology shook the scientific community, opening new avenues for stem cell research and therapy. In the original study, the introduction and overexpression of a defined set of four transcription factors (Oct4, Sox2, Klf4, c‐Myc) via virally-delivered vectors in a differentiated somatic cell, led to its forced reprogramming, resulting in the induction of pluripotency. Obtained cells resembled ESCs concerning their constitutive telomerase activity, in vivo teratoma-formation capability, ability to remain undifferentiated in an in vitro cell culture, and differentiate into cells of all three germ layers. Importantly, as iPSCs can be obtained directly from somatic cells, they do not carry the ethical burden of embryonic stem cells. Moreover, iPSCs technology allows for the generation of patient-derived therapeutic cells for autologous transplantation, hence overcoming the risk of graft rejection [152, 153]. iPSCs were already successfully differentiated into a wide variety of cells including, but not limited to, primary cells of the neural lineage: neurons [171], astrocytes [69], microglia [104], and oligodendrocytes [51].

Still, it was not before 2013 that the team led by Steve Goldman first successfully differentiated hiPSCs into oligodendrocyte progenitors. The established protocol was based on utilizing FGFs, and retinoids, followed by Shh stimulation and final activation by PDGF, IGF, and TH, and stepwise involved: step I: Formation of embryoid bodies; step II: Neuroepithelial differentiation; step III: Mechanical detachment and suspension culture; step IV: Glial differentiation, followed by gradual generation of hOPCs. hiPSC-derived hOPCs readily differentiated into astrocytes and oligodendrocytes both in vitro and in vivo. Finally, unlike iPSCs, differentiated hOPCs did not display tumorigenic potential. However, since iPSCs technology requires the cell to recapitulate all its major developmental stages, the time span of 110 days needed for successful hOPCs generation seems significant. Notably, reprogramming efficiency rose as time progressed, reaching its maximal level of 79.5 ± 8.5% on around day 150, significantly higher than for hESC-derived OPCs (45.4 ± 20.3%) [169]. Subsequent optimizing studies on iPSC-derived hOPCs included work by Douvaras and Fossati, which implemented immediate retinoids-stimulated Matrigel-based cell culture and shorter Shh exposition. As a result, it allowed for generating hOPCs more rapidly (on around day 50), with 43% efficiency on day 75 [26]. Next, a group led by Zhiguo Chen successfully reprogrammed human iPSCs to hOPCs through forced expression of virally delivered Sox10 and Olig2 TFs. Notably, around 45% of cells displayed PDGFαR^+^ phenotype after just 14 days, with O4^+^ cells present as early as day 56, signifying oligodendrocytic lineage restriction of hiPSCs-derived cells [81]. In other works, culturing human iPSCs in a dissociated monolayer and feeder-free culture system combined with FGF2, PDGF-AA, and Shh stimulation generated hOPCs from around day 85. Notably, developed OPCs could be cryopreserved, thawed, and re-plated, further facilitating their clinical use [182].

Although indirect reprogramming using iPSC technology is promising for disease modeling and creating patient-derived therapeutic cells, there are still major concerns. First, if a non-differentiated iPSC accidentally remains within the differentiated cells pool and is transplanted concomitantly, it may cause tumorigenesis due to still highly active protooncogenes [96, 118]. And second, indirectly reprogrammed cell likely retains the epigenetic memory of the mother cell used for initial iPSC generation, which could hinder its full commitment to the new phenotype [65, 72, 162].

Despite its enormous potential, iPSC technology still imposes significant risks if applied to the clinic. An ideal strategy would be delivering autologous therapeutic cells without passing through the pluripotent cell state, through direct reprogramming or transdifferentiation [29, 139].

### Directly reprogrammed somatic cells

Countless studies have already proved the successful generation of various somatic cells directly from fibroblasts via defined transcription factors (TFs), including, but not limited to, neurons [16, 164], astrocytes [17], and neural stem/precursor cells [86, 156]. Importantly, in 2013 two independent groups demonstrated the successful generation of OPCs from somatic cells by virally delivered TFs. The first team, led by Paul J. Tesar, utilized a set of eight transcription factors: Olig1, Olig2, Nkx2.2, Sox10, ST18, Nkx6.2, Myrf, and Myt1 to transdifferentiate mouse fibroblasts into OPCs with ~ 9.2% efficiency after 21 days [105]. The second group, led by Marius Wernig, used a combination of three TFs: Sox10, Olig2, and Zfp536, which proved sufficient to reprogram mouse and rat fibroblasts into OPCs with 15.6 ± 3.3% effectiveness after 3 weeks [184]. Further studies demonstrated successful generation of OPCs from astrocytes in vitro and in vivo via overexpression of a single TF: Sox10 [101] or Sox2 [28].

Although successful direct conversion of various somatic cells to GRPs is possible with virally delivered TFs, this approach still poses a significant risk of mutagenesis in the recipient cell. Therefore, other non-integrative methods were tested, including the delivery of small molecules with epigenetic activity, such as Trichostatin A (TSA), which successfully generated GRPs from mouse and human astrocytes [189]. Unfortunately, such factors are generally considered non-specific. Other approaches included simultaneous delivery of nine molecules (retinoic acid, SMER28, LDN193189, Hh-Ag1.5, CHIR99021, RG108, A83-01, Parnate, and bFGF) for conversion of mouse fibroblasts into OPCs [85]. Reprogramming efficiency was comparable to previous TF-based studies [105, 184]. Recently, based on a safe and precise, non-viral CRISPR/Cas9 system, a set of three TFs (Sox10, Olig2, and Nkx6-2) successfully reprogrammed mice fibroblasts into OPC-like cells. Although reprogramming efficiency could not be accurately quantified, the authors claim that if vector integration and transfection efficiencies were enhanced, the final transdifferentiation efficiency using CRISPR/Cas9 could exceed that of the viral-based approach [95].

To sum up, although direct reprogramming overcomes most drawbacks related to iPSC technology, further optimizations are still needed to deliver cells with clinically adequate efficiency.

### Other endogenous stem cells

Even though the directly isolated fetal and adult GRPs are proven sources for cell therapies, their initial quantity and overall expansion potential are minimal. Also, from a practical perspective, the interventional window for direct isolation is narrow, cell heterogeneity is substantial, and many ethical issues are related to obtaining material from living donors, be it fetuses or adults. Endogenous stem cells may be promising candidates for cells of origin with the molecular potential to differentiate into GRPs. Below, stem cells most commonly differentiated to GRPs with therapeutic potential are described, NSCs and mesenchymal stem cells (MSCs).

#### Neural stem cells

Neural stem cells (NSCs), also known as B-type cells, are multipotent cells derived from the radial glia of the developing CNS. They demonstrate a highly mitotic phenotype and can generate neurons, astrocytes, and oligodendrocytes via asymmetric cell division. During development, NSCs are present within the transient ventricular zone (VZ). In contrast, during adulthood, NSCs can be found within three distinct regions of the CNS: the subventricular zone of lateral ventricles (SVZ), the subgranular zone of the hippocampus (SGZ), and the olfactory epithelium (OE). NSCs are traditionally divided into mitotically quiescent B1-type cells and highly proliferative B2-type cells, giving rise to, among others, GRPs. The latter are generated before activation, which is driven by distinct cues including neurodegeneration, thus maintaining the molecular balance between differentiation and self-renewal [11, 33, 79, 125, 155].

Glial progenitors derive from primary NSCs during ontogenesis [138]. Therefore, de novo derivation of GRPs from adult NSCs relies on their stimulation with factors present during embryonic development towards glial lineages, such as FGF and Shh. In addition, protocols for generating GRPs from other sources may pass through the NSC stage [74].

It was shown that activation of Shh signaling in NSCs promotes their glial differentiation, as demonstrated by NG2^+^/Olig2^+^ OPCs [111]. Furthermore, sole EGF stimulation of NSCs spheres resulted in the generation of OPCs [193]. More modern works have shown that utilizing Shh or Smoothened Agonist (SAG), bFGF and PDGF-AA can generate OPCs from NSCs in less than a week with around 90% efficiency [82]. Recently, it was shown that inhibition of the Shh transcription factor Gli1 by GANT61 in NSCSs generated OPCs more prone towards oligodendrocytic differentiation and of a more migratory phenotype [106]. Also, overexpression of Zfp488, a factor in newly formed oligodendrocytes and involved in their maturation, in NSCs restricted them towards OPC lineage [13].

NSCs can easily give rise to GRPs after direct transplantation, as shown by multiple studies [103, 160, 180, 181]. Their glia-generating potential is so significant that “global glia replacement” was first used to describe the profound regenerative effect NSCs transplantation had on congenitally hypomyelinated shiverer mice [183].

Despite their enormous therapeutic potential, exogenous NSCs carry a similar burden as ESCs, not easily accessible tissue sources. Also, after in vivo delivery, their further development relies on endogenous molecular cues, which in a pathological state may hamper glial differentiation [132, 154].

#### Mesenchymal stem cells

Mesenchymal stem cells (MSCs) are a population of multipotent stem cells with broad differentiation potential [4, 6]. Minimal criteria defined in 2006 by the Mesenchymal and Tissue Stem Cell Committee of the International Society for Cellular Therapy qualify MSCs as cells that: (i) are plastic-adherent in standard in vitro culture conditions, (ii) express CD105, CD73, and CD90, but no CD45, CD34, CD14 or CD11b, CD79α or CD19, and HLA-DR surface antigens, (iii) can differentiate in vitro into adipocytes, osteoblasts, and chondrocytes [24].

Depending on the desired application, MSCs can be isolated from many sources, among which the placenta umbilical cord-derived Wharton’s jelly (WJ) seems particularly appealing for neural tissue regeneration [41, 115]. Indeed, as shown by Zhang et al., WJ-MSC can be safely differentiated into OPCs via neurosphere formation, followed by a distinct combination of sequentially delivered trophic factors (including FGF2 and PDGF-AA) instead of the hazardous introduction of exogenous genes. At peak point, 25.9% of differentiated OPC-like cells demonstrated PDGFR^+^ phenotype. However, despite similar morphology and phenotype, differentiated oligodendrocytes finally secreted significantly fewer neurotrophic factors than hESC-OPCs-derived oligodendrocyte counterparts, which may affect their clinical utility [191]. In other studies, sequential culturing of WJ-MSCs with: i. Insulin Transferrin Selenium and Fibronectin; ii. FGF2 and EGF; iii. FGF2 and PDGF-AA, resulted in 44.3% of cells expressing PDGFαR and 51.8% of cells expressing A2B5, two typical OPC markers [97].

Despite promising approaches, WJ is not necessarily an easily accessible source of therapeutic MSCs. However, other works have shown the successful generation of oligodendrocyte progenitors from relatively more available pools, such as human adipose-derived stem cells (hADSCs), an abundant subpopulation of mesenchymal stem cells present in human fat tissue. In one study, after multiple passages and sequentially culturing hADSCs in differentiation media consisting of, but not limited to, Shh, retinoids, neurotrophin-3 (NT3), and PDGFα, OPC-like cells with A2B5^+^ Olig2^+^ phenotype started to form with more than 90% efficiency. Unfortunately, no in vivo studies were described in this publication to verify the therapeutic potential of generated cells [34]. Finally, OPCs were differentiated from the dental pulp subpopulation of MSCs (hDPSCs) after exogenous delivery of the Olig2 gene [7] or using similar factors as in the case of hADCs [34, 100].

The potential utility of MSCs in treating glial disorders goes beyond being just the source of OPCs. For example, it was shown that MSC transplantation could activate OPCs, induce their differentiation into mature oligodendrocytes and enhance myelinization via secretion of neurotrophic factors [56] or by affecting the hosts’ immune response [9, 102]. Interestingly, even a sole conditioned medium from MSC culture can promote endogenous OPC proliferation [8].

It is, however, of note that a more precise characterization of MSC-derived oligodendrocyte precursor cells is needed. Furthermore, regardless of their source, MSCs represent a relatively heterogeneous cell pool, and their quality depends mainly on the isolation and culturing protocols, as well as the genetic background and medical history of the donor [87], which, by and large may affect their gliogenic and regenerative potential.

## Why GRP engraftment is beneficial in CNS disorders?

Given that demyelinating diseases are one of the major burdens in today’s societies, recent research is focusing on developing strategies that might improve the re-myelination process. Since deprivation of axonal myelin might also lead to neuronal degeneration [117], the loss of myelin might not only be related to demyelination but also be associated with many neurodegenerative disorders. Subsequently, it must be stressed that brain and spinal cord injuries are often accompanied by demyelination [187, 192]. Thus, it is understandable that the research is constantly evolving in response to the urgent need to discover the treatment method for CNS diseases. Recent studies more often emphasize the role of glia in both CNS physiology and pathology [70, 78, 165]. Moreover, it is more frequently stated that the degeneration of neurons is often an effect of dysfunctional glia and not the malfunctioning neuron per se [47, 75, 121]. More often, the critical role of astrocytes in CNS functioning is recognized. Those cells are known to have multiple tasks; however, in the context of direct neuron interaction, astrocytes take part in support of signal transmission, nutrition, energy supply, and homeostasis of neurons [123]. On the other hand, the second type of macroglia—oligodendrocytes—are responsible for energy supply, trophic support, ion channel organization, and myelination [2]. Due to the ability of glial progenitors to differentiate into both—astrocytes and oligodendrocytes, GRPs grafting could have beneficial outcomes in both neurodegenerative and demyelinating disorders of the CNS. Considering the latter one, unfortunately, in most cases of endogenous remyelination, even if it occurs, it seems to be not as efficient as expected. It is mainly due to the presence of only thin layers of newly generated myelin [31] that are thus not fully functional. Depending on the cause of injury, remyelination might be conducted by either resident mature oligodendrocytes that have survived the injury or glial progenitors that would migrate, proliferate and differentiate into myelinating cells [31]. The surviving mature oligodendrocytes have, however, limited migrating capacity. On the other hand, the oligodendrocyte precursors were proven to be the source of myelinating oligodendrocytes and at least a fraction of Schwann cells after demyelinating injury [190]. The number and extent of endogenous progenitors might be the limiting factors; thus, it seems that the delivery of exogenous GRPs might additionally support endogenous oligodendrocyte precursors. Indeed, several studies proved that transplantation of GRPs/OPCs might bring beneficial effects in the treatment of demyelination which will be explored in the next chapter.

On the other hand, the critical role of astroglia in most of the neuronal functions, namely in homeostasis maintenance, role in signal transmission, blood–brain barrier (BBB) regulation, etc., is that pathology of astroglia might bring disastrous effects leading to consecutive neuronal degeneration. Therefore, GRP grafting to replace the malfunctioning astroglia might also prove beneficial in treating neurodegenerative disorders like amyotrophic lateral sclerosis ALS or Huntington's disease (HD), where glia failure was recently demonstrated to be a pathological feature [163].

## Transplantation of GRPs as a therapeutic strategy for CNS diseases

**Fig. 4:**
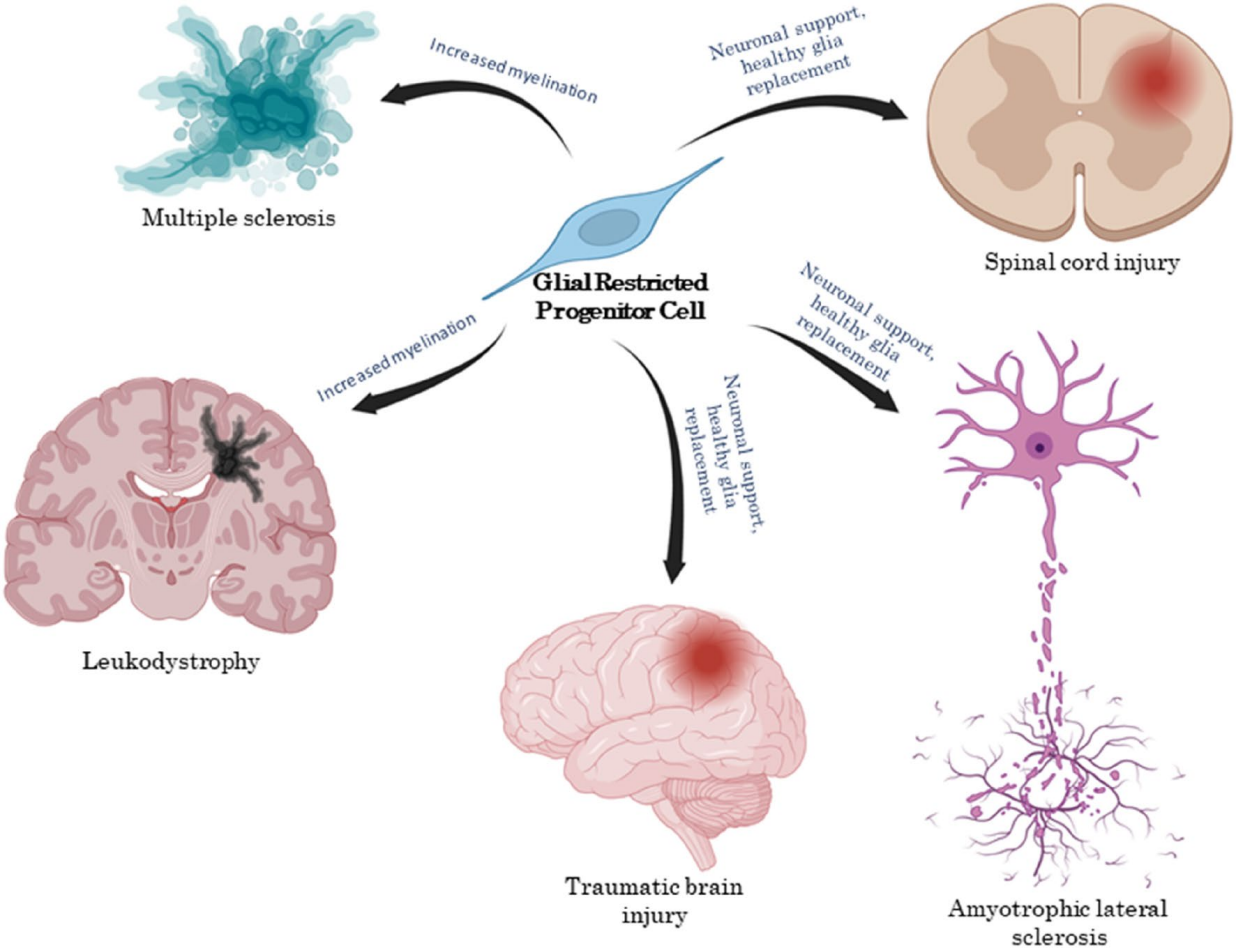
Transplantation of glial-restricted progenitor cells as a therapeutic strategy for central nervous system diseases

Stem and progenitor cell therapies are implemented in many neurological disorders, including those with white matter injuries. Central white matter diseases are linked to glial cell dysfunction leading to the loss of myelin produced by oligodendrocytes. Demyelinating disorders of CNS develop motor and cognitive deficits, which places them among the most disabling and cost-intensive neurological diseases. Therefore, in numerous therapeutic approaches, significant attention has been directed toward GRP transplantation for myelin repair and re-myelination. This strategy has been used in animal models of various demyelinating diseases such as leukodystrophies, hypomyelination, neurodegenerative diseases, and brain or spinal cord injury (Fig. 4) (Table 1).

**Table 1:** Experimental studies using GRPs and OPCs in cellular therapies for glia regeneration in different models of neurological diseases

Type of transplanted cells	Source of transplanted cells	Animal model / recipient	Number of transplanted cells	Delivery route	Observation time	Molecular outcome	Functional outcome	Human disease model	References
GRPs	I. Human fetal brain II. Human adult brain	Shiverer mice	1 × 10^5^	Intracerebral	3 months	Oligodendrocytes Astrocytes Compact myelin	Not determined	Hypomyelinating disease	[173] **
GRPs	Human fetal brain	Shiverer mice	3 × 10^5^	Intracerebral	> 1 year	Oligodendrocytes Compact myelin	Increased survival Reduced seizure frequency	Hypomyelinating disease	[174] *
GRPs	Human fetal brain	Shiverer mice	0.5 × 10^5^	Intracerebral	12 weeks	Oligodendrocytes Astrocytes Compact myelin	Not determined	Hypomyelinating disease	[142] *
GRPs	Human fetal brain	Shiverer mice	3 × 10^5^	Intracerebral	1 year	Humanization of mice brain Oligodendrocytes Astrocytes	Improved cognition Increased survival	Hypomyelinating disease	[175] **
GRPs	I. Mouse fetal brain II. Human fetal brain	Shiverer mice	2 × 10^5^	Intraventricular	I. 46 weeks II. 62 weeks	I. Early compact myelin formation with limited cell migration II. Late compact myelin formation with extensive cell migration	None	Hypomyelinating disease	[88] **
GRPs	Human fetal brain	I. Shiverer mice II. Mice with cuprizone demyelination	2 × 10^5^	Intracerebral	20 weeks	Oligodendrocytes Compact myelin Improved nerve conduction	Improved motor behaviour	Hypomyelinating disease	[176]
GRPs	Canine fetal brain and spinal cord	Shiverer mice	2 × 10^5^	Intraventricular	Up to 400 days	Partial remyelination	Extended survival	Hypomyelinating disease	[147]
OPCs	hESCs	Brain irradiated nude rats	1 × 10^6^	Intracerebral	10 weeks	Oligodendrocytes Compact myelin	Recovery from motor deficits	Hypomyelinating disease	[124]
OPCs	hiPSCs from MS patients	Shiverer mice	1 × 10^5^	Intracerebral	12–16 weeks	Myelinating oligodendrocytes	Not determined	Hypomyelinating disease	[26]
OPCs	hESCs	Shiverer mice	4 × 10^5^	Intraspinal	6 weeks	Oligodendrocytes Compact myelin	Not determined	Hypomyelinating disease	[113] *
OPCs	hiPSCs	Shiverer mice	I. 1 × 10^5^ II. 3 × 10^5^	Intracerebral	9 months	Oligodendrocytes Astrocytes Compact myelin	Increased survival	Hypomyelinating disease	[169]
OPCs overexpressing Zfp488	hiPSCs	Shiverer mice	5 × 10^5^	Intracerebral	8 weeks	Oligodendrocytes Compact myelin	Not determined	Hypomyelinating disease	[13]
OPCs	mESCs	Twitcher mice	0.2 × 10^5^	Intracerebral	~ 3 weeks	Modest myelination	None	Leukodystrophy	[71]
OPCs	Rat fetal brain	ARSA null neonate mice	1.5 × 10^5^	Intraventricular	60 days	Increased brain ARSA activity	Prevention of motor deficits	Leukodystrophy	[36]
GRPs/OPCs (sorted for: A2B5^+^, GLAST^+^, and PDGFαR^+^ populations)	Mouse fetal brain	Mice with vanishing white matter	6 × 10^5^	Intracerebral	9 months	Astrocytes Oligodendrocytes	Selectively improved motor skills	Leukodystrophy	[25]
GRPs	Rat fetal brain	SOD1 G93A rats	9 × 10^5^	Intraspinal	80 days	Astrocytes Oligodendrocytes Reduced microgliosis	Increased survival Improved motor performance	ALS	[78] *
GRPs	Human fetal brain	SOD1 G93A mice	I. 2 × 10^5^ II. 6 × 10^5^	Intraspinal	130 days	Astrocytes Oligodendrocytes	None	ALS	[77]
GRPs	Human fetal brain	SOD1 G93A mice	3 × 10^5^	Intraspinal	90 days	Retained characteristics of GRPs	Not determined	ALS	[46]
GRPs	Human fetal brain	SOD1 G93A mice	4 × 10^5^	Intracerebral	Up to 300 days	None	None	ALS	[148]
GRPs	hiPSCs	SOD1 G93A mice	8 × 10^4^	Intraspinal	up to 90 days	Astrocytes	Increased survival Improved clinical motor score	ALS	[67] *
GRPs	Mouse fetal brain	MHV-infected mice	2.4 × 10^5^	Intraspinal	33 days	Remyelination Axonal sparing	Locomotor recovery	MS	[158]
GRPs	Mouse fetal brain	MHV-infected mice	2.4 × 10^5^	Intraspinal	21 days	Remyelination without attenuated inflammatory response	Not determined	MS	[49]
OPCs	hESCs	EAE mice	1 × 10^6^	Intraventricular	15 days	Immunomodulation	Improved neurological disability score	MS	[64]
OPCs	hWJ-MSCs	EAE mice	1 × 10^6^	Intracerebroventricular	50 days	Increased remyelination	Reduced symptoms	MS	[97]
OPCs	hESCs	MS mice	1 × 10^6^	Intracerebroventricular	65 days	Reduced inflammation Reduced demyelination Reduced axonal loss	Long term disease attenuation	MS	[112]
GRPs	Mouse fetal brain	Wild-type mice (later subjected to brain trauma)	4 × 10^5^	Intracerebroventricular	16 weeks	Enhanced GRP engraftment and tissue repair after brain trauma	Not determined	Brain injury	[168]
OPCs	Human fetal brain	Ischemic stroke rats	3 × 10^5^	I. Intraventricular Ii. Intracerebral	~ 90 days	Thick myelin sheath Reduced brain structural damage	Enhanced modified neurological severity score	Brain injury	[177]
OPCs	Human fetal brain	H/I brain injury + LPS injected rats	4 × 10^5^	Intracerebroventricular	45 days	Myelinating oligodendrocytes	Improved locomotor and cognitive performance	Brain injury	[66]
OPCs	Human fetal brain	Ischemic stroke gerbils	1 × 10^6^	Intravenous	30 days	Restored myelination	Improved memory and cognition	Brain injury	[1]
OPCs	mESCs	Ischemic stroke rats	2 × 10^5^	Intraventricular	6 weeks	Myelin Apoptosis inhibition	Improved spatial learning and memory	Brain injury	[18]
OPCs	hESCs	Global cerebral ischemic rats	5 × 10^5^	Intracerebroventricular	2 weeks	Astroglia Preserved myelin integrity	Improved learning and memory	Brain injury	[57]
OPCs	Mouse adult brain	MCAO mice	6 × 10^5^	Intracerebral	Up to 14 days	Alleviated edema and infarct volume Reduced blood–brain barrier leakage	Neurological recovery promotion	Brain injury	[167]
OPCs	hESCs	Marmarou weight drop injury rat model	2 × 10^5^	Intracerebral	3 months	Ensheathing oligodendrocytes	Not determined	Brain injury	[179]
CM from GRPs	hiPSCs	MCAO rats	50 µg of total protein	Intraarterial	30 days	Enhanced angiogenesis Cytoprotection Anti-inflammatory effect	Neurological improvement	Brain injury	[137]
GRPs loaded with PAMAM	Mouse fetal spinal cord	Ischemic stroke mice	1 × 10^5^	Intracerebral	8 weeks	Improved GRPs migration and differentiation	Not determined	Brain injury	[108]
GRPs overexpressing VLA-4	Mouse fetal brain	MCAO mice	1 × 10^6^	Intraarterial	30 days	Increased efficiency of GRPs' docking Diapedesis induction	Not determined	Brain injury	[55] *
GRPs	Rat fetal spinal cord	SCI rats	3 × 10^5^	Intraspinal	3 weeks	Astrocytes Promotion of axonal growth	Not determined	SCI	[45]
GRPs	Human fetal brain	SCI rats	6 × 10^5^	Intraspinal	5 weeks	Astrocytes Axonal regeneration	Not determined	SCI	[44] *
GRPs	Human fetal brain	SCI rats	1 × 10^6^	Intraspinal	8 weeks	Mostly astrocytes Reduced glial scar	Attenuated hyperactive bladder reflexes	SCI	[58]
GRPs	Human fetal brain	SCI rats	4 × 10^5^	Intraspinal	95 days	Preserved electrophysiological conduction Myelination Astrocytes Modest generation of oligodendrocytes	None	SCI	[165] **
GRPs	Rat fetal spinal cord	SCI rats	2 × 10^5^	Intraspinal	5 weeks	Astrocytes Oligodendrocytes	Not determined	SCI	[48]
GRPs	Rat fetal brain	SCI rats	2–3 × 10^6^	Intraspinal	12 weeks	Integration within the host tissue Astrocytic and oligodendrocytic differentiation	Recovery of erectile events and locomotion functions	SCI	[114]
OPCs	hiPSCs	SCI rats	5 × 10^5^	Intraspinal	2 months	Mature oligodendrocytes Reduced cavity size Increased number of myelinated axons	Partial motor recovery	SCI	[3]
OPCs	miPSCs	SCI rats	5 × 10^6^	Intraspinal	4 weeks	Promoted myelin formation	Attenuated motor and sensory dysfunction	SCI	[188]
OPCs	hESCs	SCI rats	I. 15 × 10^5^ II. 2.5 × 10^5^	Intraspinal	8 weeks	Oligodendrocytes Improved myelination	Improved motor function	SCI	[62] *
OPCs	hiPSCs	SCI rats	5 × 10^5^	Intraspinal	12 weeks	Oligodendrocytes Neurons	None	SCI	[122]
OPCs	hESCs	SCI rats	2.4 × 10^5^—2.4 × 10^6^	Intraspinal	12 months	Neurite outgrowth Myelination	No adverse clinical observations (allodynia, toxicity, or tumor formation)	SCI	[127]
OPCs	hiPSCs	SCI rats	I. Efficacy dose: 2.4 × 10^5^ II. Maximum feasible dose: 2.4 × 10^6^	Intraspinal	4 months	Reduction in parenchymal cavitation at the injury site Increased number of myelinated axons	Improved locomotor performance	SCI	[90]
OPCs	hESCs	SCI rats	1 × 10^6^	Intraspinal	8 weeks	Neural rescue	Movement recovery	SCI	[140]
OPCs	Human fetal brain	SCI rats	I. Hemisection: 1.5 × 10^6^ II. Dorsal column: 1 × 10^6^	Intraspinal	5 weeks	Promotion of sensory and motor axonal regeneration	Not determined	SCI	[59]
OPCs	hESCs	SCI patients (clinical trial NCT02302157)	I. 2 × 10^6^ II. 1 × 10^7^ III. 2 × 10^7^	Intraparenchymal	1 year	No: enlarging mass, spinal cord damage related to the injection procedure, inflammatory lesions in the spinal cord, or masses in the VS	Moderate recovery of neurological functions	SCI (clinical trial)	[30] **
GRPs overexpressing GLT1	Rat fetal spinal cord	SCI rats	I. 6 × 10^5^ II. 9 × 10^5^	Intraspinal	5 weeks	Reduced lesion size, diaphragm denervation and diaphragm dysfunction	Not determined	SCI	[80]
OPCs overexpressing microRNA-219	hiPSCs	SCI rats	1 × 10^6^	Intraspinal	2 months	Mature oligodendrocytes	Improved locomotor activity	SCI	[107]
OPCs overexpressing MRF + Schwann cells	Rat fetal spinal cord	SCI rats	2 × 10^5^ of each cell type	Intraspinal	6 weeks	Increased myelination and tissue repair	Recovery of neurological function	SCI	[178]
OPCs overexpressing PDGF-AA	Rat fetal spinal cord	SCI rats	2 × 10^5^	Intraspinal	7 weeks	Increased myelination and tissue repair	Recovery of neurological function	SCI	[186]

*ALS* amyotrophic lateral sclerosis, *ARSA* arylsulfatase A, *CM* conditioned medium, *EAE* experimental autoimmune encephalomyelitis, *GRPs* glial-restricted progenitors, *H/I* hypoxia/ischemia, *hESC* human embryonic stem cell, *hiPSC* human induced pluripotent stem cell, *hWJ-MSC* human Wharton jelly-derived mesenchymal stem cell, *LPS* lipopolysaccharide, *MCAO* middle cerebral artery occlusion, *mESC* mouse embryonic stem cell, *MHV* mouse hepatitis virus, *miPSC* mouse induced pluripotent stem cell, *MRF* myelin regulatory factor, *MS* multiple sclerosis, *OPCs* oligodendrocyte progenitor cells, *PAMAM* hydroxyl polyamidoamine dendrimer, *PDGF-AA* platelet-derived growth factor-AA, *SCI* spinal cord injury, *SOD1* superoxide dismutase-1, *VLA4* very late antigen 4, *VS* ventricular system

*Denotes an article of special interest

**Denotes an article of outstanding interest

### Glial progenitor cell transplantation in leukodystrophies and hypomyelinating diseases

Leukodystrophies include hereditary defects in genes related to the induction of myelination, e.g., Pelizaeus-Merzbacher disease, or several inborn errors of metabolism, e.g., Sandhoffs, Tay-Sachs, Canavan’s or Krabbe’s diseases, leading to the myelin absence or loss. There are no representative experimental models of leukodystrophies. However, many current proof-of-concept studies for replacement therapy for demyelinating diseases are performed in congenitally hypomyelinated shiverer mice with a partial deletion in the myelin basic protein (MBP)-encoding gene [131]. In addition, several studies have proven that GRP transplantation into shiverer mice promoted efficient and functional myelination resulting in neurological recovery in myelin disorders (Table 1).

Over time, many populations of GRPs have been developed, either derived from fetal tissues or induced pluripotential stem cells isolated from patients. In addition, fetal hGRPs were often used as therapeutic vectors in animal models of congenital hypomyelination. To avoid xenogeneic graft rejection by the recipients, the studies were carried out on immunodeficient demyelinated mice.

Transplantation of fetal hGRPs in the forebrain of neonatal double mutant (shiverer/Rag2^−/−^) mice yielded exogenous cells spread throughout the brain and their differentiation into oligodendrocytes and astrocytes [173]. By 12 weeks after a single injection of hGRPs in neonates, compact myelin and axonal myelination were observed in the host brain. Moreover, when multiple hGRP injections were introduced into the forebrain subcortex of neonatal shiverer immunodeficient mice, a more extensive cell spreading through the white matter of transplant recipients was observed. In this case, fetal hGRPs were delivered at four sites into the corpus callosum and as a single injection into the cerebellar peduncle of shiverer/Rag2^−/−^ newborn mice. Grafted cells dispersed throughout the hosts' brain and cervical spinal cord [174]. These mice revealed the donor-derived myelin, which ensheathed host axons in the brainstem and cervical spinal cord. Moreover, multiple transplanted mice exhibited prolonged survival with neurological defect improvement compared to non-treated shiverer mice. The long-term survival of hGRP recipients enabled us to trace the process of cell treatment-associated recovery. The authors found that after 52 weeks from fetal hGRP implantation, 78% of axons in hypomyelinated shiverer mice were myelinated. Interestingly, by a year after hGRP engraftment in neonatal demyelinated immunocompromised mice, all glial progenitors and a large proportion of oligodendrocytes and astrocytes in the host brain were of human origin [175]. The same group of authors showed that fetal hGRPs sorted for high expression of PDGFαR (CD140a^+^) transplanted into shiverer mice were highly migratory and myelinated the hypomyelinated mouse brain more rapidly and efficiently than did non-sorted cells [142]. It was observed that fetal CD140a^+^ hGRPs robustly differentiate in the host brain into myelinating oligodendrocytes. By 8 weeks from transplantation, the engrafted mice exhibited significant and widespread forebrain myelination.

The other studies conducted by Walczak group demonstrated that fetal hGRPs injected bilaterally into the lateral ventricles of shiverer/Rag2^−/−^ mouse neonates displayed extensive cell migration to the brain parenchyma with a propensity to localize within the white matter structures [88]. However, hGRPs-transplanted mice revealed sparse expression of MBP, and only a few myelinated axons were observed at early-time points. The myelination became widespread at 31 weeks, but only after 62 weeks after hGRP injection the number of axons ensheathing with structurally normal myelin comparable with the pattern occurring in wild mice was found. Fetal hGRP graft significantly prolonged the survival of shiverer immunodeficient mice with a life span of over 400 days in 48% of animals.

Recently, Goldman group has shown the positive effect of GRPs transplanted into adult mice. The homozygous Rag1^−/−^ mice subjected to cuprizone demyelination were transplanted with hGRPs delivered bilaterally into the corpus callosum of 10-week-old graft recipients. The study's results revealed that hGRPs effectively dispersed throughout the forebrain of adult mice, differentiated into oligodendrocytes and myelinated demyelinated axons [176]. This data suggests that fetal hGRPs are competent to differentiate as oligodendroglia and myelinate denuded axons after their transplantation into adults, as they were able to perform remyelination when engrafted neonatally. These findings may provide a promising GRP-based treatment in patients with progressive myelin loss diseases.

Walczak and co-workers compared the effect of transplanted human or mouse GRPs (mGRPs) into shiverer/Rag2^−/−^ newborn mice and observed the graft recipients for over one year. The results of their study have shown extensive bio-distribution of hGRPs throughout the entire mouse brain, however, with a prolonged process of their differentiation into mature myelinating oligodendrocytes. In contrast, grafted mGRPs were characterized by limited migration but fast differentiation correlated with myelination observed at 18 weeks after transplantation [88]. Paradoxically, mGRP-grafted in demyelinated mice failed to extend the animal survival despite exhibiting a more pronounced presence of mature and compact myelin. Although, hGRP transplantation provided a better therapeutic effect prolonging the life span in half of the hypomyelinated leukodystrophic mice compared to mGRP injection. The authors suggested that the therapeutic mechanisms of GRP transplantation are not limited solely to the role of myelinating oligodendrocytes.

Recently, canine GRPs (cGRPs) isolated from the brain of dog fetuses were implanted intraventricularly into the double mutant immunodeficient, demyelinated neonatal shiverer mice (shiverer/Rag2^−/−^) [147]. Mapping cerebral bio-distribution of cGRPs in the host brains revealed wide dispersion of donor cells in the ventricle lining hippocampus and neighboring midbrain. Furthermore, transplantation of cGRPs resulted in visible myelination of the corpus callosum as demonstrated by MRI, immunohistochemical analysis, and electron microscopy visualization. Nevertheless, there were significant differences in myelination degree between transplanted animals, and the number of compact myelin visible during electron microscopy analysis was relatively sparse. Interestingly, shiverer mice receiving cGRPs showed improved survival in some grafted animals when compared to non-transplanted animals. The results of the studies have shown that intraventricularly injected cGRPs integrated into the brain of demyelinated mice and became functional after their transplantation. However, similarly as in the studies of Walczak group, the survival benefit of cGRP transplantation in immunodeficient shiverer mice was independent of the myelination of the corpus callosum in graft recipients.

Dooves et al. [25] compared transplantation of three different mice glial progenitor cell populations in a mice model of leukodystrophy—vanishing white matter. The mixed GPCs population was first sorted based on A2B5 expression, cells of astroglial lineage—based on GLAST expression, and OPCs based on the expression of PDGFαR. Notably, all three populations successfully integrated into the host tissue upon transplantation. No significant changes were observed in the glial fate between the different GPC populations after transplantation. Allogeneic GPC grafting procedure led to selective motor skills improvements in the hosts. Interestingly, PDGFαR^+^ cells demonstrated significantly enhanced survival in vivo compared to A2B5^+^ and GLAST^+^ cells.

Human embryonic stem cell-derived OPC transplantation was shown to remyelinate the irradiated brain and rescue behavioral deficits in rats [124]. In this model, a 50-Gy radiation dose is applied to decrease MBP expression, affecting all major white matter pathways in the brain of irradiated animals. Stereotactic bilateral injection of hESC-OPCs into the corpus callosum of nude rats 4 weeks after brain irradiation revealed donor cell migration throughout the major white matter tracts. Phenotypic analysis of the human cells surviving in the host brains demonstrated that they were mostly oligodendrocytes at various stages of maturity. In addition, the proportion of myelin-ensheathed axons was significantly increased in the grafted animals compared to the sham-operated radiation group. Finally, behavioral testing showed complete recovery of cognitive function, and the concomitant transplantation of hESC-OPCs into the cerebellum manifested additional recovery from motor deficits observed in irradiated recipients.

To translate experimental data into clinical therapeutic protocols, a sizeable pool of hGRPs would need to be generated. Therefore, the previously established method of induced pluripotent cells (iPSCs) reprogramming into neural progenitor cells (NPCs) was adapted to obtain GRPs. Wang and colleagues have found and standardized a protocol for producing bipotential astrocyte-oligodendrocyte progenitor cells from human pluripotent stem cells [169]. In addition, the myelinating potential of human iPSC-OPCs has been investigated after their transplantation in the neonatal shiverer mouse model to compare their capacity to fetal brain-derived GRPs. It was found that human iPSC-derived OPCs grafted in the same experimental conditions in immunodeficient shiverer mice maintained equal widespread migration and myelination capacity compared to human fetal GRPs. Although the transplantation of hGRPs induced myelination and led to improved survival and enhanced electrophysiological axon conduction in some shiverer/Rag2^−/−^ graft recipients, the functional recovery was not observed in the hosts.

The number of studies using rodent glial progenitors for transplantation in leukodystrophies is relatively tiny. Kuai et al. injected OPCs derived from mice ESCs into the forebrain of twitcher mice which are the animal model for human globoid cell leukodystrophy (Krabbe disease). It was shown that transplanted OPCs remained along the injection tract demonstrating limited migration abilities, and the number of donor cells significantly decreased on days 10th and 20th after injection. However, the short observation time caused by poor cell survival did not reveal significant behavioral improvements or prolongation of life span in engrafted twitcher mice [71].

The successful oligodendrocyte-based cell therapy was shown for pre-symptomatic arylsulfatase A (ARSA) null neonate mice, a murine model for human metachromatic leukodystrophy (MLD) [36]. Rat OLPs (rOPCs) engrafted into newborn MLD mice pups’ brains survived into adulthood of the hosts. Transplanted cells survived as MBP-positive post-mitotic myelinating oligodendrocytes and integrated within the white matter of adult MLD mice. OPC recipients had reduced sulfatide accumulation in the CNS, increased brain ARSA activity, and complete prevention from electrophysiological and motor deficits characteristic for untreated MLD mice.

### Glial progenitor cell transplantation in neurodegenerative diseases

In contrast to congenital demyelinated disorders, called leukodystrophies, in adults, oligodendrocyte loss contributes to neurodegenerative diseases or traumatic brain and spinal cord injuries. All of these are potential targets for GRP replacement therapy.

#### Glial progenitor cell transplantation in ALS

Amyotrophic lateral sclerosis (ALS) is a motor neuron disease resulting in progressive degeneration of the upper and lower motor neurons (MNs) in the motor cortex, brain stem, and spinal cord. Despite the relative selectivity of MN death in ALS, several experimental studies show glia involvement in the disease process. It was discovered that glial cells, mostly astrocytes, are affected by ALS pathology, and they substantially affect MN function and survival. Therefore, therapeutically targeted astrocyte replacement via transplantation of glial progenitors seems to be of great interest. Some clinical cases of ALS have been linked to various point mutations in the Cu/Zn superoxide dismutase-1 (SOD1) gene. Transgenic SOD1 G93A rodents successfully reproduce most clinical features of ALS and have been extensively used to serve as models for experimental therapeutic trials.

To assess the effect of glial progenitor transplantation, rat GRPs were injected into the cervical spinal cord of adult SOD1 G93A rats [78]. The results of this study demonstrated that transplanted GRPs survived in diseased tissue; however, the higher proportions of donor cells were located close to the injection site. Quantification of the differentiation profile of transplanted GRPs by the end-stage of the disease depicted their efficient transition into GFAP^+^ astrocytes (87.9%). A small percentage of cells also differentiated into oligodendrocytes (8.6%). Attenuation of motor neuron loss and reduced microgliosis in SOD1 G93A rat cervical spinal cord was observed in the GRP graft recipients, followed by slowed fore-limb and respiratory function declines compared to control animals.

As an extension of the previous study, the same authors investigated the effect of fetal hGRPs in the SOD1 G93A mouse model [46, 77]. hGRPs transplanted into the cervical spinal cord of immune-suppressed adult SOD1 G93A mice were found to survive in the host early in observation. The donor cells migrated both in grey and white matter and differentiated into astrocytes in the spinal cord of the graft recipients. Compared with rGRPs, the hGRPs showed much less differentiation into mature astrocytes. Moreover, the hGRP graft did not show any beneficial therapeutic effects. The lack of efficiency in motor and respiratory functional outcome may be due to the poor long-term survival of hGRPs. The authors demonstrated that hGRPs did not survive in SOD1 G93A mice until the disease end stage, despite immunosuppression.

Recently, Stanaszek and co-workers have developed an immunodeficient model of ALS (double mutant SOD1/Rag2^−/−^ mice) [89] to test the strategy of hGRPs transplantation [148]. Unfortunately, intraventricular implantation of hGRPs into SOD1/Rag2 mice neonates was not associated with improved animal survival, slowing neurodegeneration progression, or accumulation of misfolded superoxide dismutase 1. Furthermore, postmortem analysis did not reveal any surviving GRPs in the host brain at the end stage of the disease (150–200 days after transplantation).

Interestingly, glial-rich neural progenitors derived from human iPSCs (hiPSC-GRNPs) transplanted into the lumbar spinal cord of ALS model mice prolonged the life-span of graft recipients and improved clinical scores of lower limbs [67]. Transplanted cells expressed GFP reporter; thus, the authors could examine the donor cells' fate. Around 60–80% of hiPSC-GRNPs donor cells differentiated into astrocytes. Quantitative RT-PCR revealed upregulated expression of neurotrophic factors, i.e., VEGF, GDNF, and NT-3, in the lumbar spinal cord of transplanted mice. These factors were both of donor and host origin, which suggests that rather than just being direct, the neuroprotective role of hiPSC-GRNPs in motor neuron loss in SOD1 G93A mice might also be demonstrated indirectly.

#### Glial progenitor cell transplantation in MS disorders

Glia progenitor transplantation aims not always to replace affected cells but to modulate the microenvironment to improve oligodendrocyte maturation and repair the white matter. It was shown by transplantation studies in the experimental autoimmune encephalomyelitis (EAE), an animal model for multiple sclerosis (MS) where grafted cells released immunomodulatory factors that improved the disease symptoms [126].

Multiple sclerosis is an autoimmune disease caused by inflammatory attacks against myelin in the CNS. Previous reports have shown the cell-intrinsic loss of myelination competence by endogenous GRPs as the basis for remyelination failure in progressive MS disorders [109, 110].

Transplanting glial-committed progenitor cells into a viral model of MS-induced remyelination in mice chronically infected with mouse hepatitis virus (MHV). Injection of mGRPs into the spinal cord of MHV-infected mice resulted in widespread remyelination and axonal sparing within the ventral and lateral columns compared to non-transplanted animals [158]. Furthermore, mGRP grafting contributed to behavioral improvement in locomotor abilities, whereas control MHV-infected mice remained completely paralyzed. Interestingly, Hardison et al. study demonstrated that mGRP-mediated remyelination was not the result of inflammation evoked by virus-specific T cells [49]. Instead, it suggests that remyelination can occur within the inflammatory microenvironment. Indeed, several studies indicate that inflammation enhances transplanted cells' survival and migration.

Recent findings reveal that OPCs undergo a state change in MS and lose the ability to differentiate and replace lost oligodendrocytes [19]. These observations led to using exogenous glial progenitors in re-myelinating therapies to repair MS lesions.

The behavior and myelinogenic properties of transplanted glial progenitors have been demonstrated in different animal models of MS. Kim and colleagues have shown that OPCs derived from human embryonic cells transplanted in the ventricles of EAE mice revealed a significant improvement in neurological scores compared to non-transplanted animals. The positive results of transplanted OPCs are probably related to their immunomodulatory effect since the restriction of infiltrating CD45 cells was noted within the subarachnoid space in graft recipients [64]. Similarly, transplantation of OPCs derived from human Wharton’s jelly MSCs into the brain ventricles of mice with EAE after acute relapse of the symptoms significantly reduced the clinical signs of the disease [97]. In addition, histological sections from the corpus callosum of OPCs transplanted mice have shown the attenuation of demyelination severity observed in vehicle-infused control animals.

Glial progenitors inhibit the progression of EAE disease. It was found that human Olig2^+^ precursor cells derived from embryonic NSCs injected intracerebroventricularly in Biozzi AB/H mice, a chronic model of MS, reduced neuroinflammation, demyelination, and axonal loss in the cervical spinal cord, as compared to controls [112]. The donor cells were observed in the lateral ventricles and along the surface of the spinal cord 1 month after transplantation; however, they remained as glial precursors and did not express specific markers for mature oligodendrocytes. These findings suggest donor cells' bystander immunomodulatory and protective effects by attenuating demyelination and axon injury in EAE mice.

In addition to classic myelin disorders, i.e., ALS and MS, oligodendrocyte loss also contributes to other CNS diseases linked to brain or spinal cord injuries in adult patients. All of these conditions are potential targets for glial progenitor cell replacement therapy.

#### Glial progenitor cell transplantation in brain injuries

Brain injuries exhibit diverse neurobehavioral symptoms caused by the deprivation of oxygen supply leading to neuronal degeneration in the hippocampus. In addition, the white matter in the brain is affected as ischemia brings about oligodendrocyte death, myelin damage, and axon dysfunction, which are the primary cause of long-term cognitive impairment. The different experimental studies reveal that among stem cells transplanted in traumatic brain injury (TBI) disorders, glial-restricted progenitors show regenerative promise for replacing damaged cells and reducing neuroinflammation.

Transplantation of fetal mouse GRPs into lateral ventricles of newborn mice followed by TBI induced by controlled cortical impact 12 weeks after cell graft revealed colonization of donor cells in periventricular structures of the brain in adult mice [168]. In addition, MRI and histological results depicted the reduction in TBI lesion volume observed in GRP-transplanted mice in contrast to non-transplanted animals. These findings support the possibility of GRPs facilitating tissue repair by proliferating and migrating to the injury site. Furthermore, a less pronounced ratio intensity for CD45^+^ leukocytes in the local inflammatory infiltrates associated with TBI was observed in the GRP transplantation group, suggesting that the donor GRP engraftment inactivates the adaptive immune response induced by traumatic brain injury in the host brain.

A recent study by Salikhova et al. has also shown the anti-inflammatory effect of human iPSC-derived GRPs in rat middle cerebral artery occlusion (MCAO) model of ischemic stroke. The conditioned media (CM) of human iPSC-derived GPRs (GRP-CM) transplanted intra-arterially into the adult MCAO animals reduced macrophage/microglia infiltration and pro-inflammatory cytokine TNF-α gene expression and, at the same time, increased the expression of anti-inflammatory cytokine genes IL-4, IL-10, IL-13 within the brain damage area [137]. Furthermore, intra-arterial infusion of GRP-CM to MCAO stroke rat model promoted the alleviation of neurological deficits and enhanced functional recovery within 30 days of observation. Moreover, the administration of GRP-CM induced blood vessel formation in the damaged brain tissue indicating pro-angiogenic properties of GRP-secreting factors.

Wu's group investigated the long-term effect of OPC transplantation in a rat model of white matter injury established by the right common carotid artery ligation and hypoxia [177]. Human fetal OPCs injected into the lateral ventricle or white matter of 5-day-old hypoxic rat pups demonstrated thick myelin sheath and reduced structural damage in the brain compared with control animals, observed in the hosts at the age of 90 days. In addition, the transplanted rats had significantly higher modified neurological severity scores than the sham-operated group, suggesting the OPC graft's therapeutic effect.

An experimental model of periventricular leukomalacia showed the positive effect of human oligodendrocyte progenitor cell transplantation on improving neurobehavioral deficits in rats. In the study of Kim and coworkers, hOPCs were transplanted intraventricularly into 7-day-old neonatal rats subjected to hypoxia/ischemia/lipopolysaccharide (HIL) injection [66]. It was found that the donor OPCs migrated to the injured white matter area and survived more than 5 weeks in the brain of graft recipients and markedly preserved host MBP. Most importantly, transplanted animals demonstrated ameliorated locomotor and cognitive deficits of non-transplanted HIL rats.

It was reported that hOPCs infused intravenously in the aged gerbils following experimental ischemic stroke improved short-term memory and cognition deficits observed in animals after transient cerebral ischemia [1]. Interestingly, despite the donor cells not being found in the host's hippocampal parenchyma, MBP expression in this brain structure was apparently increased compared to the no transplanted ischemic groups. Furthermore, the BDNF level in the dentate gyrus of the graft recipients was much stronger than that in the vehicle-treated ischemia animals. This finding suggests that even if transplanted hOPCs do not enter the brain, they may benefit stroke disorders in term of their rehabilitation.

Deng group established the method to obtain Olig2^+^ progenitor cells derived from human embryonic stem cells (hESCs), which generate a subtype of astroglia (Olig2PC-Astros) with protective effects against ischemic brain injury [57]. These Olig2PC-Astros transplanted into the brain of adult rats after global cerebral ischemia exhibited neuroprotective effects and improved behavioral outcomes in graft recipients. Furthermore, at 2 weeks after transplantation, many donor cells survived in the ischemic brain and retained an astroglial phenotype. Moreover, the increased BDNF and MBP reactivity was observed in the hippocampal CA1 region of the host, which may contribute to the protective effects on neurons against ischemic injury.

Rodent OPCs transplanted in experimental models of brain ischemia have also provided neurotrophic benefits to surrounding impaired neural tissue. Mouse Olig2 derived from embryonic stem cells grafted into the lateral ventricles of rat pups following hypoxic-ischemic (HI) injury migrated into the parenchyma of the host brain [18]. At 6 weeks after transplantation, the donor cells were detected in the corpus callosum and the periventricular white matter. Mouse OPCs differentiated to mature oligodendrocytes and formed myelin sheath. Moreover, transplanted mOPCs were seen in the host hippocampus subgranular zone, where higher neural stem cell proliferation, Bcl-2 expression, and BDNF reactivity were observed in comparison to non-transplanted HI rats. It was found that mouse OPC transplantation reversed HI-induced spatial learning and memory deficits in graft recipients.

Recently, it was shown that OPCs isolated from rat pups and injected into adult mice after transient MCAO alleviated brain edema and infarct volume in the graft recipients [167]. Moreover, OPC transplantation decreased BBB leakage induced by its disruption evoked in ischemic brain injured rats suggesting the protection of BBB integrity through OPC graft. Furthermore, transplanted animals revealed improved neurobehavioral recovery manifested in decreased neurological scores.

Similarly, transplantation of hESC-OPCs into the cortex of nude rats subjected to diffuse traumatic axonal injury (Marmarou weight drop injury model) caused a massive migration of the donor cells to the corpus callosum and adjacent white matter accompanied by progressive maturation into oligodendrocytes that ensheathed host axons [179].

#### Glial progenitor cell transplantation in spinal cord injuries

Traumatic spinal cord injuries (SCI) involve cord compression and immediate axon and cell damage. Then the secondary degenerative changes are observed, e.g., the loss of neurons, oligodendrocytes, and myelin. Demyelination contributes to the deprivation of motor and cognitive functions, and thus, a potential therapeutic strategy involves replacing myelin-forming cells. In experimental studies, GRPs demonstrated a capacity to repair spinal cord damage, mainly through the supportive role associated with glial cells in the CNS.

Transplantation of fetal hGRPs into the contusion model of spinal cord injury in adult rats showed robust graft survival and extensive migration of donor cells at the lesion site. Fischer group studies have shown that fetal hGRP transplanted acutely into the cervical dorsal column lesion of the spinal cord in adult immunosuppressed rats survived in the injured spinal cord, filling the lesion site [44, 45]. Interestingly, the phenotypic analysis revealed that most of the animals' grafted cells differentiated into astrocytes, as characterized by the expression of GFAP. The generated permissive astrocytes supported axon growth and promoted regeneration of long ascending host sensory axons into the graft/lesion site*.* Jin et al. investigated the fate of transplanted hGRPs using athymic rats to circumvent xenograft immune issues [58]. The studies also revealed that grafted cells differentiated into glia, predominantly astrocytes, but there were few hGRPs-derived oligodendrocytes at the lesion site. It seems that transplantation of GRPs, which mainly produced astrocytes in vivo, generated a permissive environment and showed protective effects concerning secondary injury.

Walczak's group observed that fetal hGRPs transplanted into the spinal cord of adult rats with an inflammatory demyelination model expanded within the inflammatory spinal cord lesion and adopted a mature glial phenotype [165]. Moreover, transplanted rats exhibited preserved electrophysiological conduction across the spinal cord. However, the authors did not notice any behavior improvement in the focally demyelinated host after hGRP transplantation.

Few attempts have been made regarding allogeneic GRP transplantation in spinal cord injury rodent models. Han and co-workers examined the fate and migration of grafted fetal GRPs isolated from transgenic animals and transplanted into the injured spinal cord of adult rats [48]. Transplanted rGRPs survived for at least 6 weeks in the host spinal cord and differentiated along astrocytic and oligodendrocytic lineages. The donor cells migrated along white matter tracts in the injured spinal cord; however, the directed homing toward the lesion was not seen. The results of Nout et al. studies were consistent with the previous observation [114]. Rat fetal GRPs transplanted into allogeneic recipients 9 days after contusion SCI exhibited robust survival and integration into the host tissue and expressed markers for oligodendrocytes and astrocytes. Transplantation of GRPs has shown to have modest beneficial effects on some functional outcome measurements of spine cord injured rats.

Several studies revealed that human iPSC-OPCs injected into the cavity of spinal cord injured cell recipients improved neurological deficits in mice and rats following spinal cord contusion. Early intervention for SCI with iPSC-OPCs resulted in a significant increase in the number of myelinated axons and attenuated motor and sensory dysfunction in contused rats compared to control animals [3, 188].

The experimental studies with human OPC transplantation as a potential therapeutic strategy in the chronic phase of SCI in rats did not show any locomotor recovery in graft recipients; however, hOPCs survived in the host and differentiated into oligodendrocytes [62, 122].

Human embryonic stem cell-derived oligodendrocyte progenitor cell (hESC-OPC) transplantation has been tested in several rodent models of spinal cord injuries. Most therapies were performed in cervical region contusion injuries known as the frequent human traumatic SCI cases. Administration of hOPCs directly into the cervical spinal cord of nude adult rats one week after injury resulted in a significant reduction in parenchymal cavitation at the injury site and increased the number of myelinated axons [90, 127]. In addition, nude rats subjected to cervical SCI and treated with hOPCs exhibited motor behavioral recovery observed after cell transplantation. It was associated with robust engraftment of donor cells within and around the injury site and promoting neurite outgrowth in graft recipients.

Previously, the positive effects of hESC-OPC transplantation were also shown in immunocompetent adult rats subjected to acute SCI in the cervical model [140]. The spinal cord graft area of the hosts after cyclosporine A (CsA) treatment contained hESC-OPCs with their homogeneous distribution. The white matter of transplant recipient spinal cords comprised less demyelinated axons and more of properly-myelinated axons in contrast to the non-transplanted spinal cords. Moreover, the correlation between the histological and functional outcomes in graft hosts was observed for the proximal forelimb range of motion. These studies suggest that hESC-OPCs injected into the injured spinal cord have beneficial effects such as neuroprotection, axonal regeneration, and the improvement of contusion-affected forelimb function. Similar results were found by Jin and coworkers [59]. Human ESC-GRPs transplanted acutely into the spinal cord lesioned rats suppressed with cyclosporine A (CsA) modified the injury site and enhanced sensory and motor axonal growth in graft recipients.

Preclinical efficacy and safety data of a human embryonic cell-delivered oligodendrocyte progenitor cell therapy (LCTOPC1; previously known as AST-OPC1) supported a phase I/IIa clinical trial testing these cells in patients with subacute cervical spinal cord injury (NCT02302157). The study was designed as an open-label, dose-escalation, multi-center clinical trial initiated in 2014 and completed in 2021. The intra-parenchymal injection of LCTOPC1 into the spinal cord at the site of injury between 21 and 42 days after the insult was safe, and at 1-year follow-up, 96% of patients (21/22) recovered one or more levels of neurological function on at least one side of the body [30].

To conclude, accumulating evidence suggests that exogenous GRPs are promising candidates for transplantation therapy and repair of CNS functions in various demyelinating diseases such as leukodystrophies, neurodegenerative diseases, and brain or spinal cord injuries. Furthermore, it was shown that grafted glial progenitor cells survive in the neural tissue, differentiate into mature cells, promote myelination in the brain and spinal cord regions, and improve motor and sensory functions in CNS disorders. In this consent, GRPs that fulfill diverse beneficial requirements to treat distinct neurological diseases could be applied in a clinical setting (Table 1).

#### Other neurological disorders

While we have detailed above an extensive research on GRPs on several disorders, this approach can have wider ramifications. While Parkinson’s disease is strongly associated with neuronal cell death, surprisingly many genes linked to this pathology are expressed extensively in glial cells [60]. It calls for re-visiting pathology, and pointing to glia replacement as a foreseeable approach to therapy of Parkinson's disease. We have also a similar situation with Alzheimer's disease [10]. Chemobrain is another pathology in which glia is a victim but might become also a cure [166]. Glia are also considered villains of brain aging and their replacement might also contribute to rejuvenation [136, 185]. It is likely that virtually any brain disease might be linked with glia, which makes glia replacement or modulation a viable strategy to have a positive impact far beyond a few diseases in which GRPs are currently extensively investigated.

## Genetic and pharmacological enhancement of GRPs’ regenerative potential

As proved in the previous paragraphs, GRPs have the potential to show beneficial effects in the treatment of various CNS disorders, starting from demyelinating to neurodegenerative diseases. Unfortunately, in most cases of GRP transplantation, the outcome was only partial improvement. Therefore, some scientists began to develop modification methods to improve the therapeutic properties of transplanted cells (Table 1).

Two major problems related to cell therapy that require solving are the inadequate cell delivery methods and insufficient homing of grafted cells in the host’s niche. The promising cell delivery method into the brain is the intra-arterial (IA) route [5]. However, the size and the number of transplanted cells might be the limiting factors. Recently, Walczak's group performed an interesting approach where mGRPs were transfected with very late antigen 4 (VLA-4) to increase the number of infused cells capable of entering the brain parenchyma after IA delivery. VLA-4 and its ligand-vascular cell adhesion molecule 1 (VCAM-1) are responsible for leukocyte trafficking through the vessels. It seems that transfection of GRPs with VLA-4 and its following overexpression leads to increased binding to VCAM-1 and migration of the GRPs in vitro and in vivo [55].

The other report investigating GRPs' therapeutic role in spinal cord recovery attempted to overexpress glutamate transporter 1 (GLT1) in GRP-derived astrocytes. As a consequence of SCI, respiratory failure occurs, an effect of secondary degeneration leading to peripheral motoneuron (PMN) loss that innervates the diaphragm. During SCI, secondary injury results from excitotoxicity caused by glutamate clearance failure. In large part, the astrocytes are responsible for glutamate homeostasis. To diminish the cytotoxic effect occurring after SCI, Li and co-workers transplanted rat GRPs that overexpress GLT1, specifically in the astrocyte population. Such an approach resulted in the reduction of the lesion area and the preservation of diaphragm innervation and functioning. Nevertheless, there was no improvement in the forelimb grip strength in transplanted animals [80].

Positive results in SCI treatment were obtained after co-transplantation of Schwann cells with rat OPCs that overexpressed myelin gene regulatory factor (MRF). MRF is implicated in the maturation of oligodendrocytes and myelination of CNS. It must be stressed that therapeutic effects of increased myelination, reduced lesion, and recovery of some of the locomotor functions were obtained only if MRF overexpressing OPCs were co-transplanted with SCs. It seems that MRF stimulated rat OPCs into the differentiation towards myelinating oligodendrocytes in vivo. However, the MRF-overexpressing OPCs transplanted alone did not bring noticeable results [178].

Some genetic regulators like short non-coding RNA like miRNAs are also considered when it comes to the genetic manipulation of OPCs. miR-219 is responsible for the regulation of oligodendrocyte development. The overexpression of miR-219 in human OPCs transplanted in the SCI model resulted in reduced cavity size and improved functional recovery in rats. The outcome suggests that the better therapeutic result of OPC modification is somewhat related to the orientation of OPCs into mature, myelinating phenotype rather than the promotion of the survival of grafted cells [107].

Similarly, the overexpression of PDGF-AA by OPC brought beneficial results in SCI treatment. PDGF-AA is known to impact OPC proliferation and differentiation into oligodendrocytes. Yao and co-workers used spinal cord transplantation of modified rat OPCs for the treatment of SCI rats. It was proved that rats transplanted with OPCs overexpressing PDGF-AA had improved locomotor functions and diminished lesion volume compared to rats transplanted with non-modified OPCs. The positive outcome resulted from increased proliferation, improved survival, and differentiation of transplanted cells, followed by greater myelination [186].

A slightly different approach was undertaken to improve the survival of transplanted GRPs in the asphyxia mice model. Mouse GRPs were either pre-treated or co-transplanted with hydroxyl polyamidoamine dendrimer (PAMAM) conjugated with N-acetyl cysteine (NAC). NAC was previously proven to have anti-inflammatory and anti-oxidative functions. Both NAC dendrimer preconditioning of GRPs and transplantation of GRPs with NAC-dendrimers injected intraperitoneally improved migration and survival of grafted cells [108].

To summarize, GRP modification brought some positive results in terms of increasing the therapeutic properties of cells (Table 1). Nevertheless, there are still some shortcomings in the general outcome and more investigation needs to be performed before transferring GRP research into the clinic.

As demonstrated in Table 1, numerous experimental studies using GRPs/OPCs in cellular therapies for glia regeneration in different models of neurological diseases have been performed. In terms of cell source, the striking majority of these studies utilized cells derived from fetal sources or differentiated ESCs. Although such approaches are useful in preclinical settings, they carry a significant ethical burden, which hinders their effective clinical translation. Future cell therapies will more likely be based on reprogrammed autologous somatic cells. This trend is already reflected in the total number of clinical trials based on iPSCs (74.8%) vs. those based on ESCs (25.2%) [23]. As for the number of transplanted cells, the majority of the studies in Table 1 involved a pool in the range of 10^5^–10^6^ cells, depending on the disease model and/or host species. Such a number likely reflected the desired spatial distribution of exogenous cells after injection. Regarding the studied delivery routes for transplanted cells, most of them included intracerebral/intracerebroventricular and intraspinal route. This allowed to overcome the BBB hence reaching the CNS directly, yet is not necessarily the most feasible delivery route in a clinical setting due to extreme invasiveness of the procedure. Therefore, some preclinical studies were also based on minimally invasive, systemic administration routes. These included the intravenous route, which unfortunately might result in a pulmonary embolism, limiting the effectiveness of the treatment. An interesting alternative for a systemic route is intraarterial administration, allowing the delivery of a sufficient number of therapeutic cells with minimal invasiveness and a relatively simple protocol

## Consideration of clinical translation of GRP research

GRPs appear as magic but are still unfulfilled bullets. Once the rescue of the lifespan of mice suffering from leukoencephalopathy has been very inspiring [174]. Q Therapeutics made an effort to commercialize and translate of GRP-based therapeutics into a clinic. However, after many years of research, we still do not have any GRP-based clinical product. Many ethical, economic, and scientific factors contribute to this situation. Very robust Q Cells were procured from fetuses. Indeed, it was an excellent cell source for small animal research, but it ran into many clinical roadblocks. In its heyday and readiness for clinical translation, the changes in the political arena in the US, with a subsequent ban on elective abortions, contributed to the unfavorable climate surrounding cells of fetal origin. It might have translated to dampening investors’ enthusiasm, dramatically slowing research in this arena. The limited scalability of the fetal cell-based solution may be another roadblock from an investment perspective. There are also scientific reasons for less-than-expected efforts for clinical translation of GRP research. The most enthusiastic results were demonstrated after transplanting human cells to a relatively tiny mouse brain. At that time, the other company, Stem Cells, Inc., ran a clinical study on Pelizeaus-Merzbacher disease. While their proprietary cells were slightly different and likely earlier in the development, as they were called neural stem cells, they shared many characteristics with GRPs, including the capability of myelination. Unfortunately, this study was rather disappointing. Unlike the mouse study, which under MRI revealed widespread re-myelination of the dysmyelinated brain, the imaging studies showed potential re-myelination in a very limited area near the needle track [43], and the company was bankrupted, ultimately. A closer look into the images suggested that this re-myelination area may resemble the mouse brain's size, which is insufficient to make the difference in the human brain, which is roughly a thousand times bigger. Moreover, it was demonstrated that mouse GRPs are characterized by much more limited migration, which did not translate to any therapeutic effects in the same animal model of leukoencephalopathy [88]. Recently, the hypothesis of migratory range translating to the therapeutic benefit has been additionally supported by transplantation of canine GRPs, which demonstrated some benefit in a mouse model, so they were more potent than transplantation of mouse GRPs but less than human GRPs [147]. It should also be noted that Pelizeaus-Merzbacher disease is sporadic, so it is difficult to build a business case on it.

Therefore, there was a search for more frequent CNS diseases that could benefit from GRPs. ALS is undoubtedly one of these diseases, and some studies revealed the beneficial effects of GRPs and no impact of ALS on their gene expression profile [77]. Later on, other investigations pointed to a hostile host microenvironment limiting the survival of transplanted GRPs [145, 148]. Therefore, the limited migratory potential of GRPs has not been solved yet, and in other diseases than dysmyelination, such as ALS, the unfavorable microenvironment might be an additional roadblock. The ClinicalTrials.gov database revealed only two relevant clinical trials sponsored by Q Therapeutics for ALS and transverse myelitis, which were expected to start in 2021 but are still not recruiting patients. The website of Goldman’s lab reports launching the consortium composed of the three upstate New York schools for the clinical application of a slightly more differentiated cell population, namely oligodendrocyte precursors (https://www.urmc.rochester.edu/labs/goldman/projects/glial-progenitor-based-cell-therapy-in-myelin-dise.aspx; last accessed on October 27, 2022). Still, it has not been reflected in a record at ClinicalTrials.gov.

In summary, even if human GRPs proved miraculous in reversing a dismal prognosis in mice with leukoencephalopathy, additional steps are needed to enhance the migratory range of transplanted GRPs and shield them from the toxic host microenvironment. We also need to realize that we are witnessing massive progress in traditional gene therapies, such as AAV-based solutions for spinal muscular atrophy (SMA) [68], and CRISPR-based therapeutic [73], which have provided encouraging results in patients but so far have only been tried outside the CNS. Conversely, GRPs may have a widespread rejuvenating potential in the CNS. Furthermore, currently, we are witnessing enormous progress in ex vivo gene therapies. External stimuli activatable CAR T cells are just one example. Notably, these novel genetic approaches may open new technological boundaries, and can be readily used for GRP engineering to equip them with additional functions to empower them as therapeutics and make them ready to address challenges of human CNS diseases [98].

## Conclusions

As it was comprehensively described in this review, GRPs, or slightly more differentiated OPCs, have an enormous potential to treat at least some of the demyelinating and neurodegenerative diseases. In the areas where there is a need to demonstrate the protective, stabilizing effect, GRPs might act as homeostasis regulators facilitating neuronal survival after injuries. On the other hand, the ability to differentiate into myelinating oligodendrocytes makes them valuable candidates that could repair the damaged myelin sheaths of axons. However, there are many points that need to be addressed and some problems to be solved. First of all, re-myelination, if occurring, is mostly not as effective as de novo myelin formation; however, as it was shown, there is still much to be explored in this area. The other crucial aspect is the migration of transplanted cells at longer distances and the survival of the cells in the host environment that is not always hospitable. Therefore, GRPs transplantation in treating CNS diseases, even though it gives hope, is still a great field to explore.

## Data Availability

This is a narrative review based on published data.

## Abbreviations

AAV: Adeno-associated virus
AD: Alzheimer’s disease
ALS: Amyotrophic lateral sclerosis
APC: Astrocyte progenitor cell
ARSA: Arylsulfatase A
BBB: Blood-brain barrier
BDNF: Brain derived neurotrophic factor
bFGF2: Basic fibroblast growth factor 2
BMP: Bone morphogenetic protein
CAR: Chimeric antigen receptor
CD: Cluster of differentiation
cGRP: Canine glial-restricted progenitor
CM: Conditioned medium
CNS: Central nervous system
CRISPR/Cas9: Clustered Regularly Interspaced Short Palindromic Repeats/ CRISPR-associated protein 9
CsA: Cyclosporine A
CSPG4: Chondroitin sulfate proteoglycan type 4
EAE: Experimental autoimmune encephalomyelitis
EB: Embryoid body
EGF: Epidermal growth factor
ESC: Embryonic stem cell
FACS: Fluorescence-activated cell sorting
FGF: Fibroblast growth factor
GDA: Glial-derived astrocyte
GDNF: Glial cell line-derived neurotrophic factor
GFAP: Glial fibrillary acidic protein
GFP: Green fluorescent protein
GLT1: Glutamate transporter 1
GRNP: Glial-rich neural progenitor
GPC: Glial progenitor cell
GRP: Glial-restricted progenitor
hADSC: Human adipose-derived stem cell
HD: Huntington’s disease
hESC: Human embryonic stem cell
HFF: Human foreskin fibroblast
hGRP: Human glial-restricted progenitor
hiPSC: Human induced pluripotent stem cell
hOPC: Human oligodendrocyte progenitor cell
IGF: Insulin growth factor
IL: Interleukin
iPSC: Induced pluripotent stem cell
KD: Krabbe disease
MBP: Myelin basic protein
MCAO: Middle cerebral artery occlusion
MEF: Mouse embryonic fibroblast
mGRP: Mouse glial-restricted progenitor
MHV: Mouse hepatitis virus
MLD: Metachromatic leukodystrophy
MN: Motor neuron
MOG: Myelin oligodendrocyte glycoprotein
mOPC: Mouse oligodendrocyte progenitor cell
MRF: Myelin regulatory factor
MRI: Magnetic resonance imaging
MS: Multiple sclerosis
MSC: Mesenchymal stem cell
NAC: N-acetyl cysteine
NG2: Neural/glial antigen 2
NPC: Neural progenitor cell
NSC: Neural stem cell
NT3: Neurotrophin-3
O-2A: Oligodendrocyte-type-2-astrocyte
OE: Olfactory epithelium
OPC: Oligodendrocyte progenitor cell
PAMAM: Hydroxyl polyamidoamine dendrimer
PDGFα: Platelet-derived growth factor α
PDGFαR: Platelet-derived growth factor α receptor
PMD: Pelizaeus-Merzbacher disease
RAG: Recombination-activating gene
rOPC: Rat oligodendrocyte progenitor cell
RT-PCR: Reverse-transcriptase polymerase chain reaction
SCI: Spinal cord injury
SGZ: Subgranular zone
Shh: Sonic hedgehog
SMA: Spinal muscular atrophy
SOD1: Superoxide dismutase-1
SVZ: Subventricular zone
TBI: Traumatic brain injury
TF: Transcription factor
TH: Thyroid hormone
TNF-α: Tumor necrosis factor α
TSA: Trichostatin A
VCAM-1: Vascular cell adhesion molecule 1
VEGF: Vascular endothelial growth factor
VLA-4: Very late antigen 4
VZ: Ventricular zone
WJ: Wharton’s jelly
